# Supplementary material for: Existence of processes violating causal inequalities on time-delocalised subsystems
Source: Nat Commun. 2023 Mar 16;14:1471. doi: 10.1038/s41467-023-36893-3 (PMC10020554; doi:10.1038/s41467-023-36893-3)
Supplement: Supplementary file 1 — Supplementary Information [file 41467_2023_36893_MOESM1_ESM.pdf]

Existence of processes violating causal inequalities on time-delocalised subsystems: Supplementary information

Julian Wechs,[1,2] Cyril Branciard,[2] and Ognyan Oreshkov[1]

[1] *QuIC, Ecole Polytechnique de Bruxelles, C.P. 165, Université Libre de Bruxelles, 1050 Brussels, Belgium*

[2] *Univ. Grenoble Alpes, CNRS, Grenoble INP, Institut Néel, 38000 Grenoble, France*

(Dated: February 16, 2023)

SUPPLEMENTARY NOTE 1—TESTING OPERATIONS ON TIME-DELOCALISED SUBSYSTEMS

A. General circuits on time-delocalised subsystems

The structure of a circuit with respect to a particular choice of time-delocalised subsystems, as described for a generic circuit in Sec. "Time-delocalised subsystems and operations" of the main text, can be tested operationally [1]. Figuratively speaking, to achieve this, one "disconnects" the circuit fragment under consideration from its complement by "cutting through" its incoming and outgoing wires, and by "pulling all incoming, respectively outgoing, wires to the same time". Formally, this means that one performs additional SWAP operations, which send some additional incoming ancillary systems to the incoming wires of the fragment, and its outgoing wires to some additional outgoing ancillary systems (see Supplementary Fig. 1 for the example of a fragment considered in Fig. 2 of the main text). This results in the operations implemented by the fragment and its complement effectively taking place on these additional ancillary systems. By performing suitable time-local preparations (respectively, measurements) on the additional incoming (respectively, outgoing) ancillas, one can then perform tomography on these operations. Through such a procedure, one can thus in particular probe the operations that happen on the time-delocalised subsystems arising from a given choice of tensor product structure on the incoming and outgoing wires of the fragments, and test operationally that the circuit has a particular (generally cyclic) form when described with respect to these subsystems. In doing so, we make the assumption that the operations still act on these systems in the "non-disconnected" circuit, which is however completely reasonable—in fact, it is standard also for quantum circuits on time-local systems. Namely, probing the operations that make up a given standard circuit requires intervening around each operation (e.g. with suitable preparations and measurements so as to make tomography of the operation). It is an assumption that the practical procedure we employ for doing this does not alter the original operation, that is, the procedure can be described by a modified circuit that contains the original operation acting on the original systems but now connected to the probing operations via these systems, instead of to the operations from the original circuit. What we do here is the same, except that the systems we consider are time-delocalised.

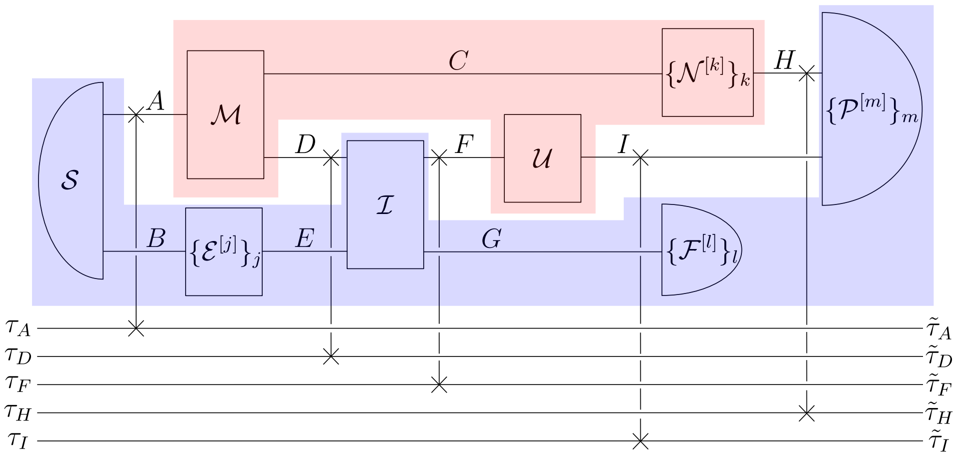

Supplementary Fig. 1. Disconnecting circuit fragments with SWAP operations. The circuit in Fig. 2(a) can effectively be decomposed into the red fragment and its blue complement, by "cutting" and "pulling to the same time" the corresponding "wires". That is, one performs SWAP operations which send some incoming ancillas τ_A, τ_F to the incoming systems A and F of the fragment, and which send the corresponding output systems of the complementary blue fragment to some outgoing ancillas $\tilde{\tau}_A$, $\tilde{\tau}_F$. Similarly, one inserts SWAP operations which send the outgoing wires D, H, I of the fragment to outgoing ancillas $\tilde{\tau}_D$, $\tilde{\tau}_H$, $\tilde{\tau}_I$, and some incoming ancillas τ_D, τ_H, τ_I to the corresponding input systems of the complementary blue fragment. This allows to test the operations implemented by the circuit fragments operationally by preparing suitable states and performing suitable measurements on the ancillas.

In the realisations of processes we considered in this work, some of the systems need to be composed in the cyclic circuit in order for the structure from the process matrix framework to emerge (namely, the systems Z, $\bar{Z}$ in the bipartite case, and the systems Y, $\bar{Y}$, Z, $\bar{Z}$, $\bar{Q}_1$ and $\bar{Q}_2'$ in the tripartite case). This raises notably the question of whether one could test the structure of the cyclic circuit in a way that leaves these systems connected, so as to probe precisely the constituents that appear in the process matrix picture. In the following, we will outline how this can be achieved for the cases considered in our paper.

B. Unitary extensions of bipartite processes on time-delocalised subsystems

Applying the general argument from Supplementary Note 1 A to the case of unitary extensions of bipartite processes studied in Methods, one could test operationally that, for any local operations U_A and U_B, and with respect to the systems P_O, A_I, A_O, A'_I, A'_O B'_I, B'_O, F_I and the time-delocalised subsystems B_I, B_O, Z and $\bar{Z}$ we identified, the bipartite circuit of Fig. 7 consists of the five operations U_A, U_B, $J_{\text{in}}^\dagger$, $J_{\text{out}}^\dagger$ and $\mathbb{1}^{Z\to\bar{Z}}$, which are connected in a cyclic manner as shown on the left-hand side of Fig. 8(c).

Once this statement has been established as an operationally verifiable fact, there exists, in particular, an operationally verifiable way to apply SWAP operations only to the incoming and outgoing time-delocalised wires B_I and B_O of U_B, so as to "cut through" and "pull to the future, respectively past" only these wires, while the time-delocalised wires Z and $\bar{Z}$ remain connected in the cyclic circuit. Namely, one can realise a "modified" temporal circuit with operations $\omega_1(U_B^{(s)})$ and $\omega_2(U_B^{(s)})$, where $U_B^{(s)}$ is related to the original U_B by $U_B^{(s)} : \mathcal{H}^{B_I B'_I \tau_{B_I} \tau_{B_O}} \to \mathcal{H}^{B_O B'_O \tilde{\tau}_{B_I} \tilde{\tau}_{B_O}}$ with $U_B^{(s)} = (\mathbb{1}^{B_O\to\tilde{\tau}_{B_O}} \otimes \mathbb{1}^{\tau_{B_O}\to B_O} \otimes \mathbb{1}^{\tilde{\tau}_{B_I} B'_O}) \cdot (U_B \otimes \mathbb{1}^{\tilde{\tau}_{B_I}\tau_{B_O}}) \cdot (\mathbb{1}^{B_I\to\tilde{\tau}_{B_I}} \otimes \mathbb{1}^{\tau_{B_I}\to B_I} \otimes \mathbb{1}^{B'_I \tau_{B_O}})$. (For the purpose of constructing the corresponding temporal circuit according to Supplementary Note 2 A, the ancillas τ_{B_I}, τ_{B_O} ($\tilde{\tau}_{B_I}$, $\tilde{\tau}_{B_O}$) can be incorporated into the incoming (outgoing) ancillary systems of U_B). By construction, in the time-delocalised description as on the left-hand side of Fig. 8(c), this modification translates to the SWAP operations which achieve the desired "disconnecting" of U_B (see Supplementary Fig. 2 for the description of the red fragment of the modified circuit in terms of the time-delocalised subsystems B_I, Z, B_O, $\bar{Z}$). Disconnecting only U_B from the cyclic circuit (and disconnecting U_A through standard, time-local SWAP operations on A_I and A_O) then in turn allows to operationally test the "coarse-grained" structure of the cyclic circuit on the right-hand side of Fig. 8(c), where $J_{\text{in}}^\dagger$, $J_{\text{out}}^\dagger$ and $\mathbb{1}^{Z\to\bar{Z}}$ are composed over the systems Z, $\bar{Z}$ so as to form the operation U that defines the process.

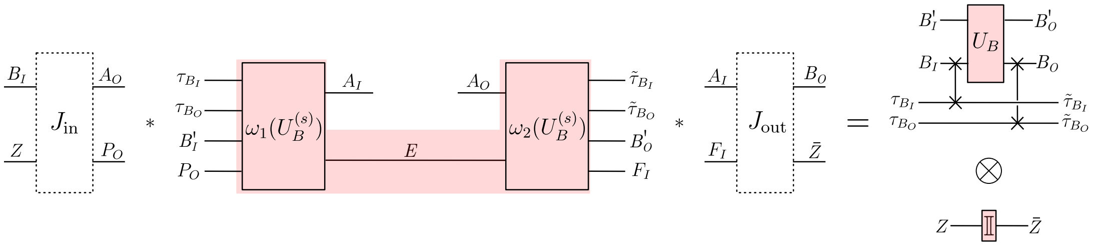

Supplementary Fig. 2. "Disconnecting" the time-delocalised wires B_I and B_O.

C. Unitary extensions of tripartite processes on time-delocalised subsystems

The tripartite case is a bit more involved, but by successively verifying a sequence of several statements that build on one another, one can similarly test that the circuit of Fig. 3 in the main text has the cyclic form on the right-hand side of Fig. 4(c) when described with respect to the systems P_O, $A_{IO}^{(\prime)}$, $B_{IO}^{(\prime)}$, $C_{IO}^{(\prime)}$, F_I. By disconnecting the red and blue fragments and applying the general procedure from Supplementary Note 1 A, one can test that, for any U_A, U_B, U_C, and with respect to the systems P_O, $A_{IO}^{(\prime)}$, $B_{IO}^{(\prime)}$, $C_{IO}^{(\prime)}$, F_I, Y, $\bar{Y}$, Z, $\bar{Z}$, $\bar{Q}_1$, $\bar{Q}'_2$, we obtain a cyclic circuit consisting of the operations U_A, U_B, $R(U_C)$ and R' (see the left-hand side of Fig. 4(c)). In this cyclic circuit, there then exists an operationally verifiable way to apply time-delocalised SWAP operations that "disconnect" precisely the time-delocalised wires C_I and Z, while the other wires remain connected. Namely, in order to achieve this, one modifies the tripartite temporal circuit by inserting some additional operations, shown in green in Supplementary Fig. 3(a), in the red fragment. In the description of the circuit with respect to P_O, $A_{IO}^{(\prime)}$, $B_{IO}^{(\prime)}$, $C_{IO}^{(\prime)}$, F_I, Y, $\bar{Y}$, Z, $\bar{Z}$, $\bar{Q}_1$, $\bar{Q}'_2$, these additional operations correspond precisely to a SWAP operation that sends the incoming ancilla τ_{C_I} into the operation $R(U_C)$ and the time-delocalised subsystem C_I to an outgoing ancilla $\tilde{\tau}_{C_I}$, and similarly for the system Z, as shown on the right-hand side of Supplementary Fig. 3(a). In an analogous way, one can modify the temporal circuit so as "disconnect" the wires C_O and $\bar{Z}$, which is shown in Supplementary Fig. 3(b). That the described modification corresponds to such SWAP operations is again an operationally verifiable statement, which can be tested by disconnecting the red and blue fragment in the modified circuit and applying the general argument from Supplementary Note 1 A, in the same way as for the tripartite circuit without the additional operations.

Once this fact has been established, by disconnecting the systems C_I, C_O, Z and $\bar{Z}$ in this way, while leaving Y, $\bar{Y}$, $\bar{Q}_1$, and $\bar{Q}'_2$ composed (and disconnecting A_I, A_O, B_I and B_O, which can be done in the same way as for B_I and B_O in the bipartite case) one can then test the structure of the cyclic circuit with respect to the systems P_O, A_I, A_O, B_I,

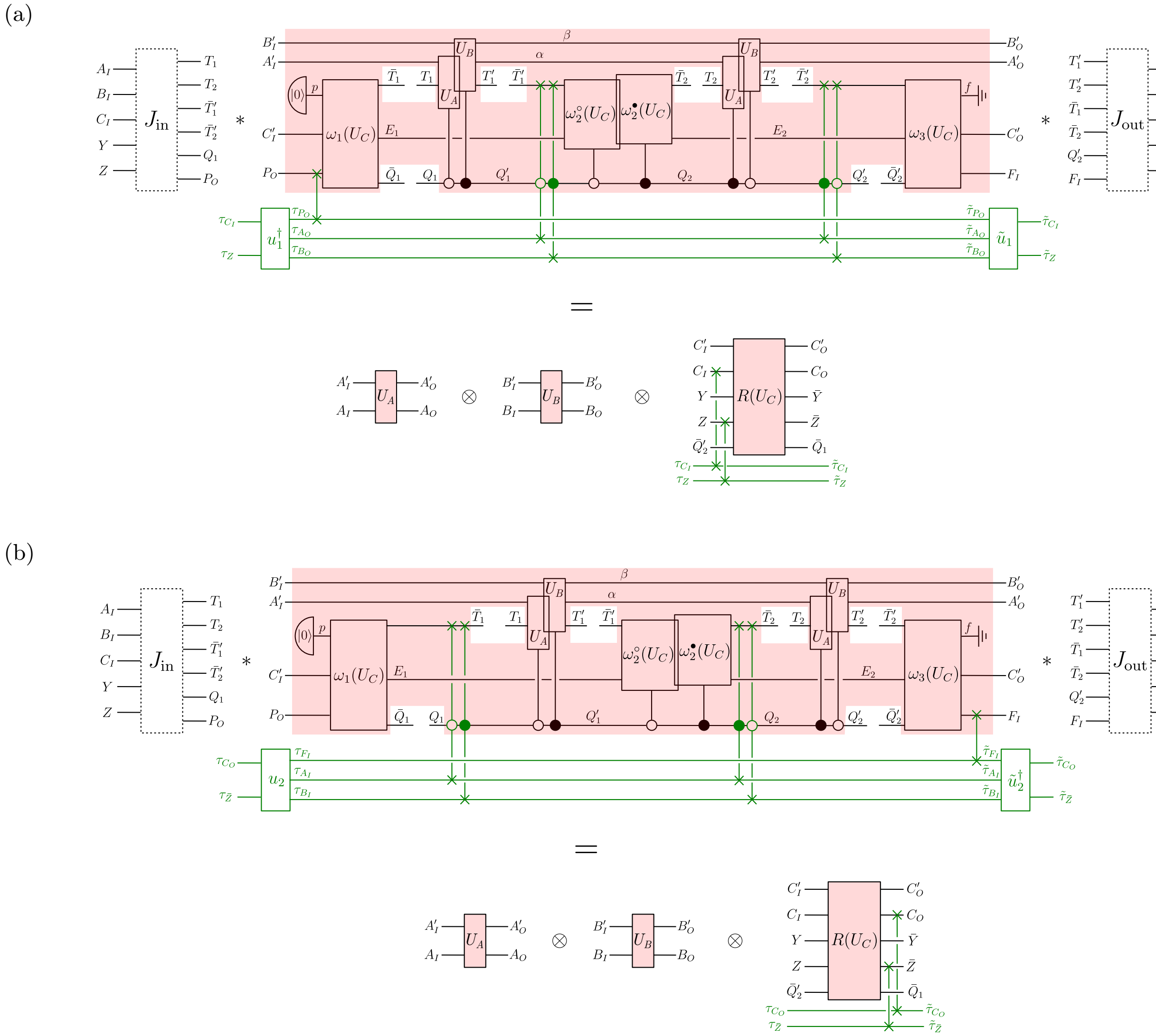

Supplementary Fig. 3. Disconnecting the time-delocalised systems C_I, Z, C_O and $\bar{Z}$. (a) Modifying the red fragment of the tripartite temporal circuit by inserting the additional operations shown in green (with $u_1 := (\mathbb{1}^{C_I \to \tau_{C_I}} \otimes \mathbb{1}^{Z \to \tau_Z}) \cdot U_1 \cdot (\mathbb{1}^{\tau_{P_O} \to P_O} \otimes \mathbb{1}^{\tau_{A_O} \to A_O} \otimes \mathbb{1}^{\tau_{B_O} \to B_O})$ and $\tilde{u}_1 := (\mathbb{1}^{C_I \to \tilde{\tau}_{C_I}} \otimes \mathbb{1}^{Z \to \tilde{\tau}_Z}) \cdot U_1 \cdot (\mathbb{1}^{\tilde{\tau}_{P_O} \to P_O} \otimes \mathbb{1}^{\tilde{\tau}_{A_O} \to A_O} \otimes \mathbb{1}^{\tilde{\tau}_{B_O} \to B_O})$, with U_1 from Eq. (24)), corresponds precisely to performing a SWAP operation that "cuts" the time-delocalised wires C_I and Z. (Note that, in order to achieve this, the second green control-SWAP operation needs to be inserted in between the identity channel relating T_2' and $\bar{T}_2'$ and the identity channel relating Q_2' and $\bar{Q}_2'$, so these identity channels need to be "shifted" against each other. But the exact placement of these identity channels in the temporal circuit is irrelevant, as long as they are between the second controlled application of U_A and U_B and the circuit operation $\omega_3(U_C)$, so that one can always choose them to be placed in that way.)
(b) The time-delocalised wires C_O and $\bar{Z}$ can be "cut" in a similar way.

B_O, C_I, C_O, F_I, Z and $\bar{Z}$. (With respect to these systems, the circuit consists of operations $U_1 : \mathcal{H}^{P_O A_O B_O} \to \mathcal{H}^{C_I Z}$, $U_2 : \mathcal{H}^{C_O \bar{Z}} \to \mathcal{H}^{A_I B_I F_I}$, $\mathbb{1}^{Z \to \bar{Z}}$, U_A, U_B and U_C (cf. Eq. (32)). Finally, once the structure of the circuit with respect to these systems has been established as an operationally verifiable fact (and, in particular, it has been established that U_C acts on the systems C_I and C_O), one can apply the analogous argument from the bipartite case to U_C in order to only "disconnect" the wires C_I and C_O and leave Z and $\bar{Z}$ connected. This in turn allows to "test" also the structure of the circuit as on the right-hand side of Fig. 4(c), where U_1, U_2 and $\mathbb{1}^{Z \to \bar{Z}}$ are connected so as to form the operation U.

SUPPLEMENTARY NOTE 2—UNITARY EXTENSIONS OF BIPARTITE PROCESSES ON TIME-DELOCALISED SUBSYSTEMS

In this Supplementary Note, we will show all technical proofs and calculations pertaining to "Unitary extensions of bipartite processes on time-delocalised subsystems" in Methods.

A. Construction of the temporal circuit

First, we show how to construct the temporal circuit shown in Fig. 7 of the main text. For any local operation U_B performed by Bob, its composition with the process vector

$$|U\rangle\rangle * |U_B\rangle\rangle =: |U_{\mathcal{G}}(\cdot, U_B)\rangle\rangle \qquad \in \mathcal{H}^{P_O A_{IO} B'_{IO} F_I} \tag{1}$$

is, mathematically, the process vector of a unitarily extended one-party process for the remaining party Alice, where the global past party has an output space $\mathcal{H}^{B'_I P_O}$, and where the global future party has an input space $\mathcal{H}^{B'_O F_I}$. (This follows from the fact that, for any unitary operation U_A performed by Alice, $|U_{\mathcal{G}}(\cdot, U_B)\rangle\rangle * |U_A\rangle\rangle = |U_{\mathcal{G}}(U_A, U_B)\rangle\rangle$ is a unitary operation from $\mathcal{H}^{A'_I B'_I P_O}$ to $\mathcal{H}^{A'_O B'_O F_I}$). It is well known [2] that the most general process of this kind is a *quantum comb* [3], and thus has a realisation as a fixed-order quantum circuit. In general, this circuit realisation can be taken to consist of two isometric operations before and after Alice's operation, which are connected by a circuit ancilla or "quantum memory" system, and whose link product is the minimal Stinespring dilation of the process matrix, with the dilating system being traced out at the end of the circuit [2–4]. Since the process here is unitary, the minimal Stinespring dilation is trivial, and the corresponding circuit isometries must be unitaries. In other words, $|U_{\mathcal{G}}(\cdot, U_B)\rangle\rangle$ can be decomposed as

$$|U_{\mathcal{G}}(\cdot, U_B)\rangle\rangle = |\omega_1(U_B)\rangle\rangle * |\omega_2(U_B)\rangle\rangle\,, \tag{2}$$

with two unitary operations $\omega_1(U_B) : \mathcal{H}^{B'_I P_O} \to \mathcal{H}^{A_I E}$ and $\omega_2(U_B) : \mathcal{H}^{A_O E} \to \mathcal{H}^{B'_O F_I}$, which depend on U_B, and with an ancillary system E (whose dimension also depends on U_B in general).

The composition $|\omega_1(U_B)\rangle\rangle * |U_A\rangle\rangle * |\omega_2(U_B)\rangle\rangle$ then describes a temporally ordered quantum circuit which by construction implements the global output operation $U_{\mathcal{G}}(U_A, U_B)$ of the process, and which consists of the three subsequent operations $\omega_1(U_B)$, $U_A : \mathcal{H}^{A_I A'_I} \to \mathcal{H}^{A_O A'_O}$ and $\omega_2(U_B)$, as shown in Fig. 7.

B. Derivation of the isomorphisms J_{in} and J_{out}

To derive the isomorphisms J_{in} and J_{out}, we first derive a mathematical relation between the systems in the process. Namely, we note that the unitary U isomorphically maps some subsystem of $A_O P_O$ to Bob's incoming system B_I, and Bob's outgoing system B_O to a subsystem of $A_I F_I$. That is, formally, U can be decomposed as $U = (\mathbb{1}^{B_I} \otimes U_2) \cdot (\mathbb{1}^{B_O} \otimes \mathbb{1}^{B_I} \otimes \mathbb{1}^{Z \to \bar{Z}}) \cdot (\mathbb{1}^{B_O} \otimes U_1)$, or, in the Choi representation,

$$|U\rangle\rangle = |U_1\rangle\rangle * |\mathbb{1}\rangle\rangle^{Z\bar{Z}} * |U_2\rangle\rangle \tag{3}$$

(see Supplementary Fig. 4) where $U_1 : \mathcal{H}^{A_O P_O} \to \mathcal{H}^{B_I Z}$ and $U_2 : \mathcal{H}^{B_O \bar{Z}} \to \mathcal{H}^{A_I F_I}$ are unitary (and the identity channel from Z to $\bar{Z}$ between the two isomorphic complementary subsystems Z and $\bar{Z}$ is introduced for later convenience).

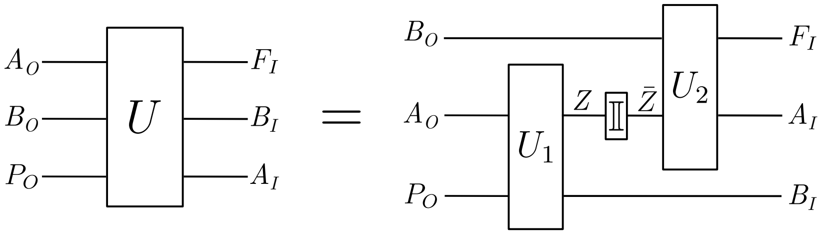

Supplementary Fig. 4. Graphical illustration of Eq. (3). The process vector describing a unitary extension of a bipartite process can be decomposed as $|U\rangle\rangle = |U_1\rangle\rangle * |\mathbb{1}\rangle\rangle^{Z\bar{Z}} * |U_2\rangle\rangle$, with unitaries $U_1 : \mathcal{H}^{A_O P_O} \to \mathcal{H}^{B_I Z}$ and $U_2 : \mathcal{H}^{B_O \bar{Z}} \to \mathcal{H}^{A_I F_I}$. The circuit on the right-hand side shows that the unitary U maps some subsystem of $A_O P_O$ to B_I, and B_O to some subsystem of $A_I F_I$.

Such a decomposition with unitary U_1 and U_2 exists for any unitarily extended bipartite process. This can be shown as follows. Consider the situation where Alice performs the specific SWAP unitary operation $S_A := \mathbb{1}^{A'_I\to A_O}\otimes\mathbb{1}^{A_I\to A'_O}$, i.e., she performs identity channels from an ancillary system A'_I to A_O and from A_I to an ancillary system A'_O which effectively "pull her output wire to the past" and "pull her input wire to the future". When the process vector is composed with that particular local operation, the result $|U_\mathcal{G}(S_A,\cdot)\rangle\!\rangle := |U\rangle\!\rangle * |S_A\rangle\!\rangle \in \mathcal{H}^{P_O A'_{IO} B_{IO} F_I}$ is now the process vector of a unitarily extended one-party process for the remaining party Bob, where the global past party has an output space $\mathcal{H}^{A'_I P_O}$, and where the global future party has an input space $\mathcal{H}^{A'_O F_I}$, and which can again be realised as a fixed-order circuit. That is, $|U_\mathcal{G}(S_A,\cdot)\rangle\!\rangle$ can be decomposed as $|U_\mathcal{G}(S_A,\cdot)\rangle\!\rangle = |\tilde{U}_1\rangle\!\rangle * |\mathbb{1}\rangle\!\rangle^{Z\bar{Z}} * |\tilde{U}_2\rangle\!\rangle$, with two circuit operations $\tilde{U}_1 : \mathcal{H}^{A'_I P_O} \to \mathcal{H}^{B_I Z}$ and $\tilde{U}_2 : \mathcal{H}^{B_O \bar{Z}} \to \mathcal{H}^{A'_O F_I}$ (which can be taken to be unitaries by the same argument as for $\omega_1(U_B)$ and $\omega_2(U_B)$ in Supplementary Note 2 A above, i.e., because the circuit realisation can be taken to achieve the minimal Stinespring dilation of the quantum comb). Since $|U\rangle\!\rangle$ can be recovered from $|U_\mathcal{G}(S_A,\cdot)\rangle\!\rangle$ through $|U\rangle\!\rangle = |U_\mathcal{G}(S_A,\cdot)\rangle\!\rangle * |\mathbb{1}\rangle\!\rangle^{A_O A'_I} * |\mathbb{1}\rangle\!\rangle^{A'_O A_I}$, one obtains the decomposition (3) for $|U\rangle\!\rangle$, with $|U_1\rangle\!\rangle := |\tilde{U}_1\rangle\!\rangle * |\mathbb{1}\rangle\!\rangle^{A_O A'_I}$ and $|U_2\rangle\!\rangle := |\tilde{U}_2\rangle\!\rangle * |\mathbb{1}\rangle\!\rangle^{A'_O A_I}$.

Up to this point, Eq. (3) is just an abstract, mathematical relation between certain systems in the process. In the circuit of Fig. 7, where A_O, P_O as well as A_I, F_I are time-local wires, this decomposition can now be taken to define an alternative description of the circuit in terms of time-delocalised subsystems. Namely, we define the isomorphisms $J_\text{in} : \mathcal{H}^{B_I Z} \to \mathcal{H}^{A_O P_O}$ and $J_\text{out} : \mathcal{H}^{A_I F_I} \to \mathcal{H}^{B_O \bar{Z}}$ simply to be the inverses of U_1 and U_2, respectively, that is, $J_\text{in} := U_1^\dagger$ and $J_\text{out} := U_2^\dagger$.

C. Changing to the description of the circuit in terms of time-delocalised subsystems

The gates of the circuit in Fig. 7 compose to the overall transformation

$$|\omega_1(U_B)\rangle\!\rangle^{P_O B'_I A_I E} * |U_A\rangle\!\rangle^{A_{IO} A'_{IO}} * |\omega_2(U_B)\rangle\!\rangle^{A_O E B'_O F_I} \tag{4}$$

from its initial systems $P_O A'_I B'_I$ to its final systems $F_I A'_O B'_O$. To change to the alternative description of the circuit in terms of time-delocalised subsystems, we start by decomposing the circuit into the blue and red circuit fragments shown in Fig. 8. That is, formally, by using the properties of the link product (i.e., its commutativity, associativity and the fact that it reduces to a tensor product for non-overlapping Hilbert spaces), we rewrite (4) as

$$\left[\,|\omega_1(U_B)\rangle\!\rangle^{P_O B'_I A_I E} * |\omega_2(U_B)\rangle\!\rangle^{A_O E B'_O F_I}\,\right] * |U_A\rangle\!\rangle^{A_{IO} A'_{IO}}\,. \tag{5}$$

The red fragment, which corresponds to the term in the first pair of square brackets, implements a unitary operation from $\mathcal{H}^{P_O B'_I A_O}$ to $\mathcal{H}^{F_I B'_O A_I}$. This unitary operation satisfies

$$\begin{aligned}
&|\omega_1(U_B)\rangle\!\rangle^{P_O B'_I A_I E} * |\omega_2(U_B)\rangle\!\rangle^{A_O E B'_O F_I} = |U_1\rangle\!\rangle^{A_O P_O B_I Z} * |\mathbb{1}\rangle\!\rangle^{Z\bar{Z}} * |U_2\rangle\!\rangle^{B_O \bar{Z} A_I F_I} * |U_B\rangle\!\rangle^{B_{IO} B'_{IO}}\\
&= |J_\text{in}^\dagger\rangle\!\rangle^{A_O P_O B_I Z} * (|U_B\rangle\!\rangle \otimes |\mathbb{1}\rangle\!\rangle^{Z\bar{Z}}) * |J_\text{out}^\dagger\rangle\!\rangle^{B_O \bar{Z} A_I F_I}\,,
\end{aligned} \tag{6}$$

where the first equality follows from combining Eqs. (1), (2) and (3) and the second equality follows from the definition of the isomorphisms J_in and J_out as the inverses of U_1 and U_2, respectively.

Therefore, when we apply J_in and J_out on the incoming and outgoing systems of the fragment, we obtain the tensor product structure

$$|J_\text{in}\rangle\!\rangle^{A_O P_O B_I Z} * \left[\,|\omega_1(U_B)\rangle\!\rangle^{P_O B'_I A_I E} * |\omega_2(U_B)\rangle\!\rangle^{A_O E B'_O F_I}\,\right] * |J_\text{out}\rangle\!\rangle^{B_O \bar{Z} A_I F_I} = |U_B\rangle\!\rangle^{B_{IO} B'_{IO}} \otimes |\mathbb{1}\rangle\!\rangle^{Z\bar{Z}}\,. \tag{7}$$

That is, the circuit fragment indeed consists of precisely one instance of U_B, which is applied locally on the time-delocalised subsystems B_I and B_O, in parallel to an identity channel from Z to $\bar{Z}$, as is shown in Fig. 8(a).

In order to describe the entire circuit in terms of the newly chosen subsystem description, we need to rewrite also the blue circuit fragment, which simply consists of U_A, in terms of this new subsystem description. To do this, we compose its incoming wire A_I with the isomorphism $J_\text{out}^\dagger = U_2$, and its outgoing wire A_O with $J_\text{in}^\dagger = U_1$ (see Fig. 8(b)).

Recomposing the two fragments in the new subsystem decomposition over the systems Z, $\bar{Z}$ then yields

$$\begin{aligned}
&[|U_B\rangle\!\rangle^{B_{IO} B'_{IO}} \otimes |\mathbb{1}\rangle\!\rangle^{Z\bar{Z}}] * [|U_2\rangle\!\rangle^{B_O \bar{Z} A_I F_I} * |U_A\rangle\!\rangle^{A_{IO} A'_{IO}} * |U_1\rangle\!\rangle^{A_O P_O B_I Z}]\\
=&[|U_1\rangle\!\rangle^{A_O P_O B_I Z} \otimes |U_2\rangle\!\rangle^{B_O \bar{Z} A_I F_I} * |\mathbb{1}\rangle\!\rangle^{Z\bar{Z}}] * |U_A\rangle\!\rangle^{A_{IO} A'_{IO}} * |U_B\rangle\!\rangle^{B_{IO} B'_{IO}}\\
=&|U\rangle\!\rangle^{P_O A_{IO} B_{IO} F_I} * |U_A\rangle\!\rangle^{A_{IO} A'_{IO}} \otimes |U_B\rangle\!\rangle^{B_{IO} B'_{IO}}\,,
\end{aligned} \tag{8}$$

as shown graphically in Fig. 8(c).

SUPPLEMENTARY NOTE 3—UNITARY EXTENSIONS OF TRIPARTITE PROCESSES ON TIME-DELOCALISED SUBSYSTEMS

In this Supplementary Note, we will show all technical proofs and calculations pertaining to "Unitary extensions of tripartite processes on time-delocalised subsystems" of the main text.

A. Construction of the temporal circuit

The bipartite proof relies crucially on the fact that the unitarily extended one-party process that one obtains by fixing Bob's operation has a particular "standard form"—namely, a circuit in which Alice's operation acts at a fixed time. This provides us with some fixed, time-local(ised) physical systems relative to which we can define the time-delocalised subsystems on which Bob's operation acts based on the mapping effected by the unitary that defines the process. For unitary extensions of bipartite processes, it was shown in Refs. [5, 6] that a similar standard form exists. Namely, all unitary extensions of bipartite processes are *variations of the quantum switch*. This result forms the basis for the tripartite construction we derive in this paper.

In Refs. [5, 6], the following characterisation was proven. For any process vector $|U\rangle\rangle \in \mathcal{H}^{P_O A_{IO} B_{IO} F_I}$ of a unitary extension of a bipartite process, the output, respectively input Hilbert spaces of the global past and future parties can be decomposed into a direct sum $\mathcal{H}^{P_O} = \mathcal{H}^{P_O^\ell} \oplus \mathcal{H}^{P_O^r}$ and $\mathcal{H}^{F_I} = \mathcal{H}^{F_I^\ell} \oplus \mathcal{H}^{F_I^r}$, such that $|U\rangle\rangle$ has the form

$$|U\rangle\rangle = |\nu_1^{A\prec B}\rangle\rangle * |\nu_2^{A\prec B}\rangle\rangle * |\nu_3^{A\prec B}\rangle\rangle + |\nu_1^{B\prec A}\rangle\rangle * |\nu_2^{B\prec A}\rangle\rangle * |\nu_3^{B\prec A}\rangle\rangle \tag{9}$$

with unitary operations $\nu_1^{A\prec B} : \mathcal{H}^{P_O^\ell} \to \mathcal{H}^{A_I} \otimes \mathcal{H}^{\lambda_1}$, $\nu_2^{A\prec B} : \mathcal{H}^{A_O} \otimes \mathcal{H}^{\lambda_1} \to \mathcal{H}^{B_I} \otimes \mathcal{H}^{\lambda_2}$, $\nu_3^{A\prec B} : \mathcal{H}^{B_O} \otimes \mathcal{H}^{\lambda_2} \to \mathcal{H}^{F_I^\ell}$, as well as unitary operations $\nu_1^{B\prec A} : \mathcal{H}^{P_O^r} \to \mathcal{H}^{B_I} \otimes \mathcal{H}^{\rho_1}$, $\nu_2^{B\prec A} : \mathcal{H}^{B_O} \otimes \mathcal{H}^{\rho_1} \to \mathcal{H}^{A_I} \otimes \mathcal{H}^{\rho_2}$, $\nu_3^{B\prec A} : \mathcal{H}^{A_O} \otimes \mathcal{H}^{\rho_2} \to \mathcal{H}^{F_I^r}$ (and conversely, any vector of the form as in Eq. (9) is the process vector of a valid unitarily extended bipartite process). In other words, any such $|U\rangle\rangle$ can be decomposed into a sum of two process vectors, the first of which describes a fixed-order circuit (i.e., a quantum comb) with the "global past" output space $\mathcal{H}^{P_O^\ell}$ and the "global future" input space $\mathcal{H}^{F_I^\ell}$, which consists of unitary circuit operations and in which U_A is applied before U_B. Similarly, the second summand in the decomposition (9) corresponds to a quantum comb with the "global past" output space $\mathcal{H}^{P_O^r}$ and the "global future" input space $\mathcal{H}^{F_I^r}$, in which U_B is applied before U_A.

First, we address a technicality regarding the dimensions of the systems in such a decomposition. Namely, the two combs in Eq. (9) generally involve ancillas of different dimensions $d_{\lambda_1} = d_{\lambda_2}$ and $d_{\rho_1} = d_{\rho_2}$ (or equivalently, global past and future spaces with different dimensions $d_{P_O^\ell} = d_{F_I^\ell}$ and $d_{P_O^r} = d_{F_I^r}$). For the construction of an alternative temporal circuit for unitarily extended bipartite circuits that we will consider below (Supplementary Fig. 5), it is however convenient to consider a process in which the dimensions in these two combs are the same. We therefore show that any unitarily extended bipartite process as in Eq. (9) can be recovered as part of a suitable "enlarged" process for which this condition on the dimension is indeed satisfied.

To define this enlarged process, we consider arbitrary "complementary" unitary operations $\nu_{1,\text{comp.}}^{A\prec B} : \mathcal{H}^{P_O^r} \to \mathcal{H}^{A_I} \otimes \mathcal{H}^{\rho_1}$, $\nu_{2,\text{comp.}}^{A\prec B} : \mathcal{H}^{A_O} \otimes \mathcal{H}^{\rho_1} \to \mathcal{H}^{B_I} \otimes \mathcal{H}^{\rho_2}$, $\nu_{3,\text{comp.}}^{A\prec B} : \mathcal{H}^{B_O} \otimes \mathcal{H}^{\rho_2} \to \mathcal{H}^{F_I^r}$, as well as $\nu_{1,\text{comp.}}^{B\prec A} : \mathcal{H}^{P_O^\ell} \to \mathcal{H}^{B_I} \otimes \mathcal{H}^{\lambda_1}$, $\nu_{2,\text{comp.}}^{B\prec A} : \mathcal{H}^{B_O} \otimes \mathcal{H}^{\lambda_1} \to \mathcal{H}^{A_I} \otimes \mathcal{H}^{\lambda_2}$ and $\nu_{3,\text{comp.}}^{B\prec A} : \mathcal{H}^{A_O} \otimes \mathcal{H}^{\lambda_2} \to \mathcal{H}^{F_I^\ell}$. We then define $\mathcal{H}^{E_1} := \mathcal{H}^{\lambda_1} \oplus \mathcal{H}^{\rho_1}$ and $\mathcal{H}^{E_2} := \mathcal{H}^{\lambda_2} \oplus \mathcal{H}^{\rho_2}$, and introduce two additional two-dimensional Hilbert spaces $\mathcal{H}^p$ (with computational basis $\{|0\rangle^p, |1\rangle^p\}$) and $\mathcal{H}^f$ (with computational basis $\{|0\rangle^f, |1\rangle^f\}$). From that, we implicitly consider the various ν unitaries above to act on the extended ancillary spaces $\mathcal{H}^{E_i}$ (by adding null contributions on the originally untouched subspaces $\mathcal{H}^{\rho_i}$ or $\mathcal{H}^{\lambda_i}$) and define the "enlarged" operations (denoted here with tildes) $\tilde{\nu}_1^{A\prec B} : \mathcal{H}^{\tilde{P}_O^\ell} \to \mathcal{H}^{A_I} \otimes \mathcal{H}^{E_1}$, with $\mathcal{H}^{\tilde{P}_O^\ell} := \mathcal{H}^{P_O^\ell} \otimes \text{span}\{|0\rangle^p\} \oplus \mathcal{H}^{P_O^r} \otimes \text{span}\{|1\rangle^p\}$, as

$$\tilde{\nu}_1^{A\prec B} := \nu_1^{A\prec B} \otimes \langle 0|^p + \nu_{1,\text{comp.}}^{A\prec B} \otimes \langle 1|^p\,, \tag{10}$$

$\tilde{\nu}_2^{A\prec B} : \mathcal{H}^{A_O} \otimes \mathcal{H}^{E_1} \to \mathcal{H}^{B_I} \otimes \mathcal{H}^{E_2}$ as

$$\tilde{\nu}_2^{A\prec B} := \nu_2^{A\prec B} + \nu_{2,\text{comp.}}^{A\prec B} \tag{11}$$

and $\tilde{\nu}_3^{A\prec B} : \mathcal{H}^{B_O} \otimes \mathcal{H}^{E_2} \to \mathcal{H}^{\tilde{F}_I^\ell}$, with $\mathcal{H}^{\tilde{F}_I^\ell} := \mathcal{H}^{F_I^\ell} \otimes \text{span}\{|0\rangle^f\} \oplus \mathcal{H}^{F_I^r} \otimes \text{span}\{|1\rangle^f\}$, as

$$\tilde{\nu}_3^{A\prec B} := \nu_3^{A\prec B} \otimes |0\rangle^f + \nu_{3,\text{comp.}}^{A\prec B} \otimes |1\rangle^f\,. \tag{12}$$

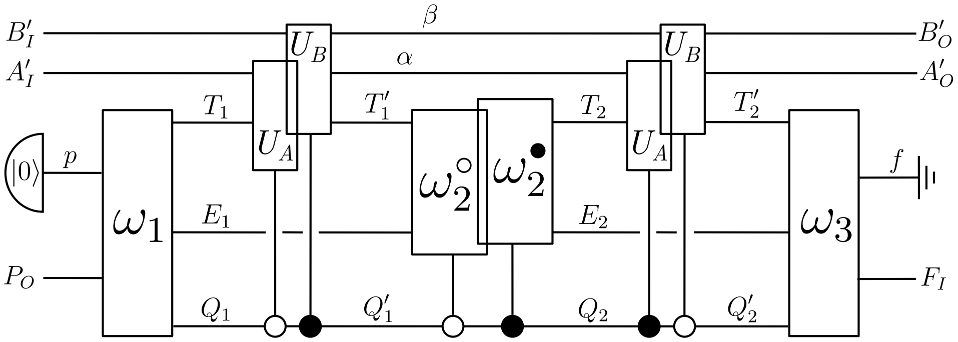

Supplementary Fig. 5. Unitary extensions of bipartite processes can be realised as "variations of the quantum switch".

Similarly, we define $\tilde{\nu}_1^{B\prec A}: \mathcal{H}^{\tilde{P}_O^r} \to \mathcal{H}^{B_I} \otimes \mathcal{H}^{E_1}$, with $\mathcal{H}^{\tilde{P}_O^r} := \mathcal{H}^{P_O^r} \otimes \mathrm{span}\{|0\rangle^p\} \oplus \mathcal{H}^{P_O^\ell} \otimes \mathrm{span}\{|1\rangle^p\}$, as

$$\tilde{\nu}_1^{B\prec A} := \nu_1^{B\prec A} \otimes \langle 0|^p + \nu_{1,\mathrm{comp.}}^{B\prec A} \otimes \langle 1|^p\,, \tag{13}$$

$\tilde{\nu}_2^{B\prec A}: \mathcal{H}^{B_O} \otimes \mathcal{H}^{E_1} \to \mathcal{H}^{A_I} \otimes \mathcal{H}^{E_2}$ as

$$\tilde{\nu}_2^{B\prec A} := \nu_2^{B\prec A} + \nu_{2,\mathrm{comp.}}^{B\prec A} \tag{14}$$

and $\tilde{\nu}_3^{B\prec A}: \mathcal{H}^{A_O} \otimes \mathcal{H}^{E_2} \to \mathcal{H}^{\tilde{F}_I^r}$, with $\mathcal{H}^{\tilde{F}_I^r} := \mathcal{H}^{F_I^r} \otimes \mathrm{span}\{|0\rangle^f\} \oplus \mathcal{H}^{F_I^\ell} \otimes \mathrm{span}\{|1\rangle^f\}$, as

$$\tilde{\nu}_3^{B\prec A} := \nu_3^{B\prec A} \otimes |0\rangle^f + \nu_{3,\mathrm{comp.}}^{B\prec A} \otimes |1\rangle^f\,. \tag{15}$$

These thus defined enlarged operations act unitarily on input and output Hilbert spaces that all have the same dimension. They can be combined to an enlarged process vector

$$|\tilde{U}\rangle\!\rangle := |\tilde{\nu}_1^{A\prec B}\rangle\!\rangle * |\tilde{\nu}_2^{A\prec B}\rangle\!\rangle * |\tilde{\nu}_3^{A\prec B}\rangle\!\rangle + |\tilde{\nu}_1^{B\prec A}\rangle\!\rangle * |\tilde{\nu}_2^{B\prec A}\rangle\!\rangle * |\tilde{\nu}_3^{B\prec A}\rangle\!\rangle\,, \tag{16}$$

with a "global past" party that has two output systems P_O and p (with the corresponding Hilbert space $\mathcal{H}^{P_O p}$ consisting of the two orthogonal subspaces of the same dimensions $\mathcal{H}^{\tilde{P}_O^\ell}$, corresponding to the "$A \prec B$ branch", and $\mathcal{H}^{\tilde{P}_O^r}$, corresponding to the "$B \prec A$ branch"), and a "global future" party that has two input systems F_I and f (with the corresponding Hilbert space $\mathcal{H}^{F_I f}$ consisting of the two orthogonal subspaces of the same dimensions $\mathcal{H}^{\tilde{F}_I^\ell}$, corresponding to the "$A \prec B$ branch", and $\mathcal{H}^{\tilde{F}_I^r}$, corresponding to the "$B \prec A$ branch"). The enlarged process vector has the desired property that the circuit ancillas are the same in each coherent branch (namely, E_1 and E_2). The original process is recovered when the state $|0\rangle^p$ is prepared in the output subsystem p of the global past party, and the subsystem f of the global future party is discarded, i.e. $|U\rangle\!\rangle\langle\!\langle U| = \mathrm{Tr}_f[|0\rangle\langle 0|^p * |\tilde{U}\rangle\!\rangle\langle\!\langle \tilde{U}|]$ (note that all link products between the original and "complementary" operations evaluate to zero, since they are composed over orthogonal subspaces of the circuit ancillas E_1 and E_2; when inputting the initial state $|0\rangle^p$ the subsystem f is also guaranteed to end up in the state $|0\rangle^f$, so that discarding f above is then in fact equivalent to projecting it onto $|0\rangle^f$, and does not introduce any decoherence).

With this in place, we now construct an alternative temporal circuit for unitary extensions of bipartite processes (different from the one in Fig. 7), which will provide the basis for the tripartite generalisation. This temporal circuit is shown in Supplementary Fig. 5.

The circuit receives an input state in the output space $\mathcal{H}^{P_O}$ of the "global past" party, as well as the fixed state $|0\rangle^p$ in the space $\mathcal{H}^p$ (see above). A unitary operation $\omega_1: \mathcal{H}^{P_O p} \to \mathcal{H}^{T_1 E_1 Q_1}$ given by

$$\omega_1 = \left[(\mathbb{1}^{E_1} \otimes \mathbb{1}^{A_I \to T_1}) \cdot \tilde{\nu}_1^{A\prec B}\right] \otimes |0\rangle^{Q_1} + \left[(\mathbb{1}^{E_1} \otimes \mathbb{1}^{B_I \to T_1}) \cdot \tilde{\nu}_1^{B\prec A}\right] \otimes |1\rangle^{Q_1} \tag{17}$$

(with $\tilde{\nu}_1^{A\prec B}$ from Eq. (10) and $\tilde{\nu}_1^{B\prec A}$ from Eq. (13), each being implicitly and trivially extended to act on the whole space $\mathcal{H}^{P_O p} = \mathcal{H}^{\tilde{P}_O^\ell} \oplus \mathcal{H}^{\tilde{P}_O^r}$ rather than on just one of the subspaces) then takes these systems to a d-dimensional "target system" T_1, a two-dimensional "control system" Q_1 (with computational basis $\{|0\rangle^{Q_1}, |1\rangle^{Q_1}\}$) and a circuit ancilla E_1. After that, a time-local instance of Alice's operation U_A or Bob's operation U_B is applied to the target system, coherently conditioned on the control system being in the states $|0\rangle^{Q_1}$, or $|1\rangle^{Q_1}$, respectively; that is, formally,

the circuit applies the unitary operation

$$[(\mathbb{1}^{A_O\to T_1'}\otimes\mathbb{1}^{A_O'\to\alpha})\cdot U_A\cdot(\mathbb{1}^{T_1\to A_I}\otimes\mathbb{1}^{A_I'})]\otimes\mathbb{1}^{B_I'\to\beta}\otimes|0\rangle^{Q_1'}\langle 0|^{Q_1}$$
$$+[(\mathbb{1}^{B_O\to T_1'}\otimes\mathbb{1}^{B_O'\to\beta})\cdot U_B\cdot(\mathbb{1}^{T_1\to B_I}\otimes\mathbb{1}^{B_I'})]\otimes\mathbb{1}^{A_I'\to\alpha}\otimes|1\rangle^{Q_1'}\langle 1|^{Q_1}\,, \tag{18}$$

where $\mathcal{H}^{\alpha}$ is of the same dimension as Alice's local ancillary spaces $\mathcal{H}^{A_I'}$ and $\mathcal{H}^{A_O'}$, and $\mathcal{H}^{\beta}$ is of the same dimension as Bob's local ancillary spaces $\mathcal{H}^{B_I'}$ and $\mathcal{H}^{B_O'}$. The circuit then proceeds with a coherently controlled application of a unitary operation $\omega_2^{\circ}:\mathcal{H}^{T_1'E_1}\to\mathcal{H}^{T_2E_2}$ or $\omega_2^{\bullet}:\mathcal{H}^{T_1'E_1}\to\mathcal{H}^{T_2E_2}$. That is, it applies the unitary operation

$$\omega_2^{\circ}\otimes|0\rangle^{Q_2}\langle 0|^{Q_1'}+\omega_2^{\bullet}\otimes|1\rangle^{Q_2}\langle 1|^{Q_1'}\,. \tag{19}$$

with

$$\omega_2^{\circ}=(\mathbb{1}^{E_2}\otimes\mathbb{1}^{B_I\to T_2})\cdot\tilde{\nu}_2^{A\prec B}\cdot(\mathbb{1}^{T_1'\to A_O}\otimes\mathbb{1}^{E_1}), \tag{20}$$

as well as

$$\omega_2^{\bullet}=(\mathbb{1}^{E_2}\otimes\mathbb{1}^{A_I\to T_2})\cdot\tilde{\nu}_2^{B\prec A}\cdot(\mathbb{1}^{T_1'\to B_O}\otimes\mathbb{1}^{E_1}). \tag{21}$$

(with $\tilde{\nu}_2^{A\prec B}$ from Eq. (11) and $\tilde{\nu}_2^{B\prec A}$ from Eq. (14)). Then follows the second controlled "time-local" application of Alice's and Bob's operations to the target system, which is given by

$$[(\mathbb{1}^{B_O\to T_2'}\otimes\mathbb{1}^{B_O'})\cdot U_B\cdot(\mathbb{1}^{T_2\to B_I}\otimes\mathbb{1}^{\beta\to B_I'})]\otimes\mathbb{1}^{\alpha\to A_O'}\otimes|0\rangle^{Q_2'}\langle 0|^{Q_2}$$
$$+[(\mathbb{1}^{A_O\to T_2'}\otimes\mathbb{1}^{A_O'})\cdot U_A\cdot(\mathbb{1}^{T_2\to A_I}\otimes\mathbb{1}^{\alpha\to A_I'})]\otimes\mathbb{1}^{\beta\to B_O'}\otimes|1\rangle^{Q_2'}\langle 1|^{Q_2}\,. \tag{22}$$

Finally, the circuit terminates with a unitary operation $\omega_3:\mathcal{H}^{T_2'E_2Q_2'}\to\mathcal{H}^{F_If}$ given by

$$\omega_3=\left[\tilde{\nu}_3^{A\prec B}\cdot(\mathbb{1}^{E_2}\otimes\mathbb{1}^{T_2'\to B_O})\right]\otimes\langle 0|^{Q_2'}+\left[\tilde{\nu}_3^{B\prec A}\cdot(\mathbb{1}^{E_2}\otimes\mathbb{1}^{T_2'\to A_O})\right]\otimes\langle 1|^{Q_2'} \tag{23}$$

(with $\tilde{\nu}_3^{A\prec B}$ from Eq. (12) and $\tilde{\nu}_3^{B\prec A}$ from Eq. (15), again implicitly considering that they each output a state in the whole space $\mathcal{H}^{F_If}=\mathcal{H}^{\tilde{F}_I^{\ell}}\oplus\mathcal{H}^{\tilde{F}_I^{r}}$ rather than in just one of the subspaces) that takes the target, control and ancillary systems to the input Hilbert space of the "global future" party and the system $\mathcal{H}^{f}$, which is then traced out (or equivalently projected onto $|0\rangle^{f}$, see above).

Such a circuit is an example of a *quantum circuit with quantum control of causal order* [7], and similarly to the case of fixed-order quantum circuits, the operations ω_1, ω_2°, $\omega_2^{\bullet}$ and ω_3 can be constructed explicitly from the process matrix.

Let us now consider a unitary extension of a tripartite process, described by a process vector $|U\rangle\!\rangle\in\mathcal{H}^{P_OA_{IO}B_{IO}C_{IO}F_I}$. When the process vector is composed with a fixed unitary operation $|U_C\rangle\!\rangle^{C_{IO}C_{IO}'}$ for Charlie, the result $|U_{\mathcal{G}}(\cdot,\cdot,U_C)\rangle\!\rangle:=|U\rangle\!\rangle*|U_C\rangle\!\rangle^{C_{IO}C_{IO}'}$ is, mathematically, a process vector describing a unitary extension of a bipartite process (for the remaining parties Alice and Bob) with a "global past" party whose output space is $\mathcal{H}^{P_OC_I'}$ and a "global future" party whose input space is $\mathcal{H}^{F_IC_O'}$, and which can be implemented as a "variation of the quantum switch", as presented just above. For the full tripartite process, one can therefore find a temporal description consisting of a "variation of the quantum switch", where the circuit operations ω_1, ω_2°, $\omega_2^{\bullet}$ and ω_3 and the dimensions of E_1 and E_2 depend on U_C. To finally get to the tripartite circuit of Fig. 3, for later convenience, we introduce identity channels on the systems T_1, T_2, T_1', T_2', Q_1 and Q_2', which relate the respective system to a copy of it, denoted by the same label with a "bar" superscript.

B. Derivation of the isomorphisms J_{in} and J_{out}

Note that the decomposition in Eq. (3) generalises straightforwardly to an arbitrary numbers of parties. That is, for a unitary extension of some multipartite process, it is still true that the unitary maps isomorphically the output system of a given party to a subsystem of the input systems of all other parties and the global future system, and similarly, it maps a subsystem of the output of all but one party and the global past to the input of that remaining party. For instance, for a unitary extension of a tripartite process, one can formally decompose its process vector $|U\rangle\!\rangle\in\mathcal{H}^{P_OA_{IO}B_{IO}C_{IO}F_I}$ as

$$|U\rangle\!\rangle=|U_1\rangle\!\rangle*|\mathbb{1}\rangle\!\rangle^{Z\bar{Z}}*|U_2\rangle\!\rangle\,, \tag{24}$$

with unitaries $U_1 : \mathcal{H}^{P_O A_O B_O} \to \mathcal{H}^{C_I Z}$ and $U_2 : \mathcal{H}^{C_O \bar{Z}} \to \mathcal{H}^{A_I B_I F_I}$ (cf. Supplementary Fig. 6). This follows from precisely the same argument as in the bipartite case, by considering the reduced process with one operation for Charlie, obtained by transferring Alice's and Bob's input (output) wires to the global future (past) via SWAP operations and identity channels. (The fact that this abstract, mathematical decomposition generalises to the multipartite case was

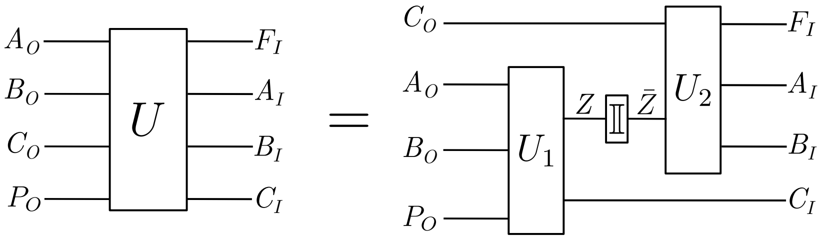

Supplementary Fig. 6. Graphical representation of Eq. (24). The process vector of a tripartite unitary process can be decomposed as $|U\rangle\rangle = |U_1\rangle\rangle * |\mathbb{1}\rangle\rangle^{Z\bar{Z}} * |U_2\rangle\rangle$, with unitaries $U_1 : \mathcal{H}^{A_O B_O P_O} \to \mathcal{H}^{C_I Z}$ and $U_2 : \mathcal{H}^{C_O \bar{Z}} \to \mathcal{H}^{A_I B_I F_I}$. The circuit on the right-hand side shows that the unitary U maps some subsystem of $A_O B_O P_O$ to C_I, and C_O to some subsystem of $A_I B_I F_I$.

also noted in Ref. [8].) From that decomposition, we then define the isomorphisms $J_{\text{in}} : \mathcal{H}^{A_I B_I C_I Y Z} \to \mathcal{H}^{T_1 T_2 \bar{T}_1' \bar{T}_2' Q_1 P_O}$ and $J_{\text{out}} : \mathcal{H}^{T_1' T_2' \bar{T}_1 \bar{T}_2 Q_2' F_I} \to \mathcal{H}^{A_O B_O C_O \bar{Y} \bar{Z}}$ to be

$$\begin{aligned} J_{\text{in}} =& \mathbb{1}^{A_I \to T_1} \otimes \mathbb{1}^{B_I \to T_2} \otimes [(\mathbb{1}^{A_O \to \bar{T}_1'} \otimes \mathbb{1}^{B_O \to \bar{T}_2'} \otimes \mathbb{1}^{P_O}) \, U_1^\dagger] \otimes |0\rangle^{Q_1} \langle 0|^Y \\ &+ \mathbb{1}^{B_I \to T_1} \otimes \mathbb{1}^{A_I \to T_2} \otimes [(\mathbb{1}^{B_O \to \bar{T}_1'} \otimes \mathbb{1}^{A_O \to \bar{T}_2'} \otimes \mathbb{1}^{P_O}) \, U_1^\dagger] \otimes |1\rangle^{Q_1} \langle 1|^Y \end{aligned} \tag{25}$$

and

$$\begin{aligned} J_{\text{out}} =& \mathbb{1}^{T_1' \to A_O} \otimes \mathbb{1}^{T_2' \to B_O} \otimes [U_2^\dagger \, (\mathbb{1}^{\bar{T}_1 \to A_I} \otimes \mathbb{1}^{\bar{T}_2 \to B_I} \otimes \mathbb{1}^{F_I})] \otimes |0\rangle^{\bar{Y}} \langle 0|^{Q_2'} \\ &+ \mathbb{1}^{T_1' \to B_O} \otimes \mathbb{1}^{T_2' \to A_O} \otimes [U_2^\dagger \, (\mathbb{1}^{\bar{T}_1 \to B_I} \otimes \mathbb{1}^{\bar{T}_2 \to A_I} \otimes \mathbb{1}^{F_I})] \otimes |1\rangle^{\bar{Y}} \langle 1|^{Q_2'} . \end{aligned} \tag{26}$$

J_{in} can be represented graphically as in Supplementary Fig. 7(a), and J_{out} as in Supplementary Fig. 7(b).

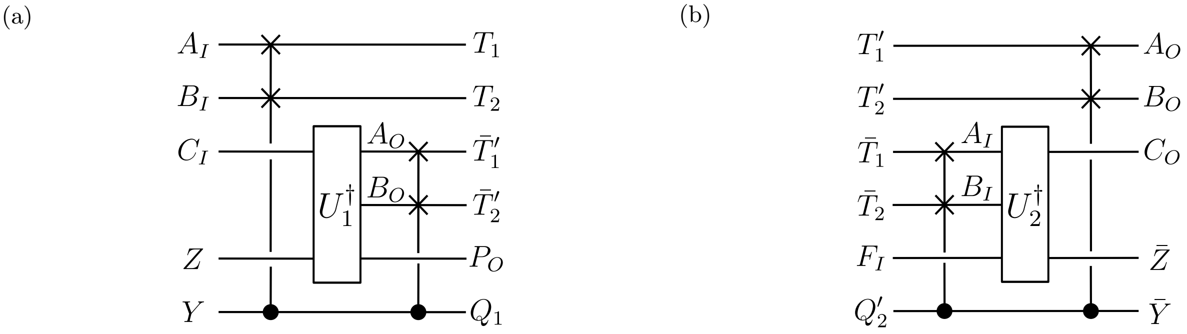

Supplementary Fig. 7. Graphical representation of the isomorphisms J_{in} and J_{out}. (a) Graphical representation of the isomorphism J_{in}, with which the incoming systems of the red fragment of Fig. 4(a) (and the outgoing systems of the blue fragment in Fig. 4(b)) are composed so as to move to the description in terms of time-delocalised subsystems. Alice's input system A_I is mapped to the "target" incoming system T_1, and Bob's input system B_I is mapped to the "target" incoming system T_2, or vice versa, conditionally on whether the additional system Y (whose computational basis state is transmitted to the control system Q_1) is in the state $|0\rangle$ or $|1\rangle$. The unitary $U_1^\dagger$ from the abstract decomposition in Eq. (24) (see also Supplementary Fig. 6) maps Charlie's input system C_I and the additional system Z to $A_O B_O P_O$, with A_O then being mapped to $\bar{T}_1'$ and B_O to $\bar{T}_2'$, or vice versa, conditionally on the state of the control system.
(b) Graphical representation of the isomorphism J_{out}, with which the outgoing systems in the red fragment of Fig. 4(a) (and the incoming systems of the blue fragment in Fig. 4(b)) are composed. The "target" outgoing system T_1' is mapped to Alice's output system A_O, and the "target" outgoing system T_2' to Bob's output system B_O, or vice versa, depending on whether the control system Q_2' (whose computational basis state is transmitted to the additional system $\bar{Y}$) is in the state $|0\rangle$ or $|1\rangle$. The "target" system $\bar{T}_1$ is mapped to the system A_I, and $\bar{T}_2$ to B_I, or vice versa, conditionally on the state of the control system, and $A_I B_I F_I$ is mapped to Charlie's output system C_O and the additional system $\bar{Z}$ by the unitary $U_2^\dagger$ from the abstract decomposition in Eq. (24).

C. Description of the circuit in terms of time-delocalised subsystems

To describe the circuit in terms of the chosen time-delocalised subsystems, we consider the red circuit fragment in Fig. 4(a), which implements a quantum operation $\Phi_1(U_A, U_B, U_C)$ from $\mathcal{H}^{A'_I B'_I C'_I P_O T_1 T_2 \bar{T}'_1 \bar{T}'_2 Q_1 \bar{Q}'_2}$ to $\mathcal{H}^{A'_O B'_O C'_O F_I \bar{T}_1 \bar{T}_2 T'_1 T'_2 \bar{Q}_1 Q'_2}$, described in the pure Choi representation by

$$
\begin{aligned}
&|\Phi_1(U_A, U_B, U_C)\rangle\!\rangle \\
&= |\omega_1^{[0]}(U_C)\rangle\!\rangle^{P_O C'_I \bar{T}_1 E_1 \bar{Q}_1} * \Big(|U_A^{(1)}\rangle\!\rangle^{T_1 A'_I T'_1 \alpha} \otimes |\mathbb{1}\rangle\!\rangle^{B'_I \beta} \otimes |0\rangle^{Q_1} \otimes |0\rangle^{Q'_1} + |U_B^{(1)}\rangle\!\rangle^{T_1 B'_I T'_1 \beta} \otimes |\mathbb{1}\rangle\!\rangle^{A'_I \alpha} \otimes |1\rangle^{Q_1} \otimes |1\rangle^{Q'_1} \Big) \\
&\qquad\qquad * \Big(|\omega_2^{\circ}(U_C)\rangle\!\rangle^{\bar{T}'_1 E_1 \bar{T}_2 E_2} \otimes |0\rangle^{Q'_1} \otimes |0\rangle^{Q_2} + |\omega_2^{\bullet}(U_C)\rangle\!\rangle^{\bar{T}'_1 E_1 \bar{T}_2 E_2} \otimes |1\rangle^{Q'_1} \otimes |1\rangle^{Q_2} \Big) \\
&\qquad * \Big(|U_B^{(2)}\rangle\!\rangle^{T_2 \beta T'_2 B'_O} \otimes |\mathbb{1}\rangle\!\rangle^{\alpha A'_O} \otimes |0\rangle^{Q_2} \otimes |0\rangle^{Q'_2} + |U_A^{(2)}\rangle\!\rangle^{T_2 \alpha T'_2 A'_O} \otimes |\mathbb{1}\rangle\!\rangle^{\beta B'_O} \otimes |1\rangle^{Q_2} \otimes |1\rangle^{Q'_2} \Big) * |\omega_3^{[0]}(U_C)\rangle\!\rangle^{\bar{T}'_2 E_2 \bar{Q}'_2 F_I C'_O} \\
&= |\omega_1^{[0]}(U_C)\rangle\!\rangle^{P_O C'_I \bar{T}_1 E_1 \bar{Q}_1} * \Big(\big[|U_A^{(1)}\rangle\!\rangle^{T_1 A'_I T'_1 \alpha} * |\mathbb{1}\rangle\!\rangle^{\alpha A'_O} \big] \otimes |\omega_2^{\circ}(U_C)\rangle\!\rangle^{\bar{T}'_1 E_1 \bar{T}_2 E_2} \otimes \big[|\mathbb{1}\rangle\!\rangle^{B'_I \beta} * |U_B^{(2)}\rangle\!\rangle^{T_2 \beta T'_2 B'_O} \big] \otimes |0\rangle^{Q_1} \otimes |0\rangle^{Q'_2} \\
&+ \big[|U_B^{(1)}\rangle\!\rangle^{T_1 B'_I T'_1 \beta} * |\mathbb{1}\rangle\!\rangle^{\beta B'_O} \big] \otimes |\omega_2^{\bullet}(U_C)\rangle\!\rangle^{\bar{T}'_1 E_1 \bar{T}_2 E_2} \otimes \big[|\mathbb{1}\rangle\!\rangle^{A'_I \alpha} * |U_A^{(2)}\rangle\!\rangle^{T_2 \alpha T'_2 A'_O} \big] \otimes |1\rangle^{Q_1} \otimes |1\rangle^{Q'_2} \Big) * |\omega_3^{[0]}(U_C)\rangle\!\rangle^{\bar{T}'_2 E_2 \bar{Q}'_2 F_I C'_O}
\end{aligned} \tag{27}
$$

with the shorthand notations $|\omega_1^{[0]}(U_C)\rangle\!\rangle^{P_O C'_I \bar{T}_1 E_1 \bar{Q}_1} := |0\rangle^p * |\omega_1(U_C)\rangle\!\rangle^{P_O p C'_I \bar{T}_1 E_1 \bar{Q}_1}$, $|U_A^{(1)}\rangle\!\rangle^{T_1 A'_I T'_1 \alpha} := |\mathbb{1}\rangle\!\rangle^{T_1 A_I} * |U_A\rangle\!\rangle^{A_{IO} A'_{IO}} * (|\mathbb{1}\rangle\!\rangle^{A_O T'_1} \otimes |\mathbb{1}\rangle\!\rangle^{A'_O \alpha})$, $|U_B^{(1)}\rangle\!\rangle^{T_1 B'_I T'_1 \beta} := |\mathbb{1}\rangle\!\rangle^{T_1 B_I} * |U_B\rangle\!\rangle^{B_{IO} B'_{IO}} * (|\mathbb{1}\rangle\!\rangle^{B_O T'_1} \otimes |\mathbb{1}\rangle\!\rangle^{B'_O \beta})$, $|U_A^{(2)}\rangle\!\rangle^{T_2 \alpha T'_2 A'_O} := (|\mathbb{1}\rangle\!\rangle^{T_2 A_I} \otimes |\mathbb{1}\rangle\!\rangle^{\alpha A'_I}) * |U_A\rangle\!\rangle^{A_{IO} A'_{IO}} * |\mathbb{1}\rangle\!\rangle^{A_O T'_2}$, $|U_B^{(2)}\rangle\!\rangle^{T_2 \beta T'_2 B'_O} := (|\mathbb{1}\rangle\!\rangle^{T_2 B_I} \otimes |\mathbb{1}\rangle\!\rangle^{\beta B'_I}) * |U_B\rangle\!\rangle^{B_{IO} B'_{IO}} * |\mathbb{1}\rangle\!\rangle^{B_O T'_2}$ and $|\omega_3^{[0]}(U_C)\rangle\!\rangle^{\bar{T}'_2 E_2 \bar{Q}'_2 F_I C'_O} := |\omega_3(U_C)\rangle\!\rangle^{\bar{T}'_2 E_2 \bar{Q}'_2 F_I f C'_O} * |0\rangle^f$. In terms of the time-delocalised systems defined above, the operation implemented by the circuit fragment reads

$$
|J_{\text{in}}\rangle\!\rangle * |\Phi_1(U_A, U_B, U_C)\rangle\!\rangle * |J_{\text{out}}\rangle\!\rangle = |U_A\rangle\!\rangle^{A_{IO} A'_{IO}} \otimes |U_B\rangle\!\rangle^{B_{IO} B'_{IO}} \otimes |R(U_C)\rangle\!\rangle^{C_{IO} C'_{IO} Y \bar{Y} Z \bar{Z} \bar{Q}_1 \bar{Q}'_2} \tag{28}
$$

(see Fig. 4(a)), where we denote by $|R(U_C)\rangle\!\rangle$ the pure Choi representation of the operation $R(U_C) : \mathcal{H}^{C'_I C_I Y Z \bar{Q}'_2} \to \mathcal{H}^{C'_O C_O \bar{Y} \bar{Z} \bar{Q}_1}$ happening in parallel to U_A and U_B, which is given by

$$
\begin{aligned}
&|R(U_C)\rangle\!\rangle^{C_{IO} C'_{IO} Y \bar{Y} Z \bar{Z} \bar{Q}_1 \bar{Q}'_2} \\
&= \big(|U_1^\dagger\rangle\!\rangle^{C_I Z P_O A_O B_O} * [|\mathbb{1}\rangle\!\rangle^{A_O \bar{T}'_1} \otimes |\mathbb{1}\rangle\!\rangle^{B_O \bar{T}'_2}] \big) * |\omega_1^{[0]}(U_C)\rangle\!\rangle^{P_O C'_I \bar{T}_1 E_1 \bar{Q}_1} * |\omega_2^{\circ}(U_C)\rangle\!\rangle^{\bar{T}'_1 E_1 \bar{T}_2 E_2} * |\omega_3^{[0]}(U_C)\rangle\!\rangle^{\bar{T}'_2 E_2 \bar{Q}'_2 F_I C'_O} \\
&\qquad\qquad * \big([|\mathbb{1}\rangle\!\rangle^{\bar{T}_1 A_I} \otimes |\mathbb{1}\rangle\!\rangle^{\bar{T}_2 B_I}] * |U_2^\dagger\rangle\!\rangle^{A_I B_I F_I C_O \bar{Z}} \big) \otimes |0\rangle^{\bar{Y}} \otimes |0\rangle^{Y} \\
&+ \big(|U_1^\dagger\rangle\!\rangle^{C_I Z P_O A_O B_O} * [|\mathbb{1}\rangle\!\rangle^{B_O \bar{T}'_1} \otimes |\mathbb{1}\rangle\!\rangle^{A_O \bar{T}'_2}] \big) * |\omega_1^{[0]}(U_C)\rangle\!\rangle^{P_O C'_I \bar{T}_1 E_1 \bar{Q}_1} * |\omega_2^{\bullet}(U_C)\rangle\!\rangle^{\bar{T}'_1 E_1 \bar{T}_2 E_2} * |\omega_3^{[0]}(U_C)\rangle\!\rangle^{\bar{T}'_2 E_2 \bar{Q}'_2 F_I C'_O} \\
&\qquad\qquad * \big([|\mathbb{1}\rangle\!\rangle^{\bar{T}_1 B_I} \otimes |\mathbb{1}\rangle\!\rangle^{\bar{T}_2 A_I}] * |U_2^\dagger\rangle\!\rangle^{A_I B_I F_I C_O \bar{Z}} \big) \otimes |1\rangle^{\bar{Y}} \otimes |1\rangle^{Y} .
\end{aligned} \tag{29}
$$

$R(U_C)$ is represented graphically in Supplementary Fig. 8.

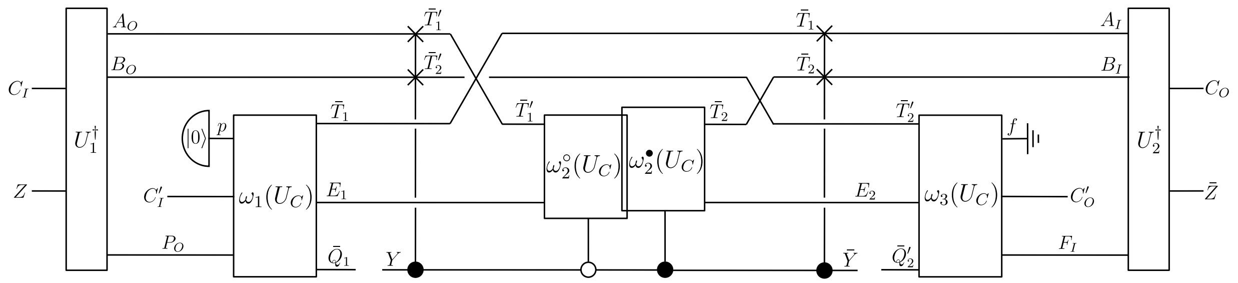

Supplementary Fig. 8. Circuit representation of $R(U_C)$, obtained by linking the red circuit fragment in Fig. 4(a) with the circuits of J_{in} and J_{out} from Supplementary Fig. 7, and "factorising out" U_A and U_B.

To complete the rewriting of the circuit in terms of these time-delocalised subsystems, we also need to compose the operation implemented by the complementary blue fragment, which consists of the identity channels relating the

systems with and without the “bar” superscripts, with precisely the inverse of the isomorphisms J_{in} and J_{out}. We obtain

$$\begin{aligned} |R'\rangle\rangle &:= |J_{\text{out}}^\dagger\rangle\rangle * \big[|\mathbb{1}\rangle\rangle^{\bar{T}_1 T_1} \otimes |\mathbb{1}\rangle\rangle^{\bar{T}_2 T_2} \otimes |\mathbb{1}\rangle\rangle^{T'_1 \bar{T}'_1} \otimes |\mathbb{1}\rangle\rangle^{T'_2 \bar{T}'_2} \otimes |\mathbb{1}\rangle\rangle^{\bar{Q}_1 Q_1} \otimes |\mathbb{1}\rangle\rangle^{Q'_2 \bar{Q}'_2} \big] * |J_{\text{in}}^\dagger\rangle\rangle \\ &= |U_2\rangle\rangle^{C_O \bar{Z} A_I B_I F_I} \otimes |U_1\rangle\rangle^{P_O A_O B_O C_I Z} \otimes \big[|0\rangle^{\bar{Y}} \otimes |0\rangle^{\bar{Q}'_2} \otimes |0\rangle^{\bar{Q}_1} \otimes |0\rangle^{Y} + |1\rangle^{\bar{Y}} \otimes |1\rangle^{\bar{Q}'_2} \otimes |1\rangle^{\bar{Q}_1} \otimes |1\rangle^{Y} \big] \\ &\quad + |(\mathfrak{S}^{A_I B_I} \otimes \mathbb{1}^{F_I}) U_2\rangle\rangle^{C_O \bar{Z} A_I B_I F_I} \otimes |U_1(\mathfrak{S}^{A_O B_O} \otimes \mathbb{1}^{P_O})\rangle\rangle^{P_O A_O B_O C_I Z} \\ &\quad \otimes \big[|0\rangle^{\bar{Y}} \otimes |0\rangle^{\bar{Q}'_2} \otimes |1\rangle^{\bar{Q}_1} \otimes |1\rangle^{Y} + |1\rangle^{\bar{Y}} \otimes |1\rangle^{\bar{Q}'_2} \otimes |0\rangle^{\bar{Q}_1} \otimes |0\rangle^{Y} \big] \end{aligned} \tag{30}$$

(see Fig. 4(b)), where $\mathfrak{S}^{A_I B_I} := \mathbb{1}^{A_I \to B_I} \otimes \mathbb{1}^{B_I \to A_I}$ and $\mathfrak{S}^{A_O B_O} := \mathbb{1}^{A_O \to B_O} \otimes \mathbb{1}^{B_O \to A_O}$ are SWAP operations. The operation R' is represented graphically in Supplementary Fig. 9.

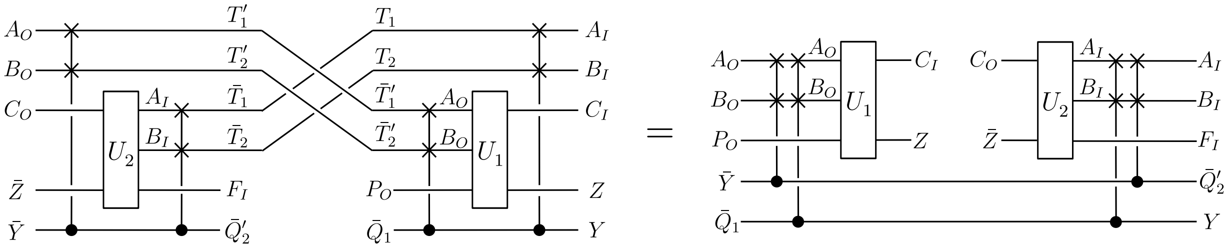

Supplementary Fig. 9. Circuit representation of R', obtained by linking the reversed circuits of J_{in} and J_{out} from Fig. 7 with the blue circuit fragment in Fig. 4(b). One easily sees, as in Eq. (30), that if the control states in systems $\bar{Y}\bar{Q}'_2$ and $\bar{Q}_1 Y$ are the same, then the controlled SWAP operations don't have any effect, while if the control states are different, these effectively swap the A and B systems. (Note that the latter situation, and the corresponding terms in Eq. (30), get cancelled when linking R' to $R(U_C)$.)

Finally, we want to recompose the operations $|R(U_C)\rangle\rangle$ and $|R'\rangle\rangle$ over the systems Y, $\bar{Y}$, Z, $\bar{Z}$, $\bar{Q}_1$, $\bar{Q}'_2$ (but not over the systems C_I and C_O). Inserting the explicit expressions for ω_1, ω_2°, $\omega_2^\bullet$ and ω_3 (Eqs. (17), (20), (21) and (23), respectively), taking the link product of the right-hand sides of Eq. (29) and Eq. (30) and evaluating the link products over Y, $\bar{Y}$, $\bar{Q}_1$, $\bar{Q}'_2$, $\bar{T}_1$, $\bar{T}_2$, $\bar{T}'_1$, $\bar{T}'_2$, yields

$$\begin{aligned} &|R(U_C)\rangle\rangle * |R'\rangle\rangle \\ &= |U_1\rangle\rangle^{P_O A_O B_O C_I Z} * \Big(|U_1^\dagger\rangle\rangle^{C_I Z P_O A_O B_O} * \big[|\nu_1^{A \prec B}(U_C)\rangle\rangle^{P_O C'_I A_I E_1} * |\nu_2^{A \prec B}(U_C)\rangle\rangle^{A_O E_1 B_I E_2} * |\nu_3^{A \prec B}(U_C)\rangle\rangle^{B_O E_2 F_I C'_O} \\ &\quad + |\nu_1^{B \prec A}(U_C)\rangle\rangle^{P_O C'_I B_I E_1} * |\nu_2^{B \prec A}(U_C)\rangle\rangle^{B_O E_1 A_I E_2} * |\nu_3^{B \prec A}(U_C)\rangle\rangle^{A_O E_2 F_I C'_O} \big] * |U_2^\dagger\rangle\rangle^{A_I B_I F_I C_O \bar{Z}} \Big) * |U_2\rangle\rangle^{C_O \bar{Z} A_I B_I F_I} . \end{aligned} \tag{31}$$

We then note that the term in the square bracket on the right-hand side of Eq. (31) is precisely $|U_{\mathcal{G}}(\cdot,\cdot,U_C)\rangle\rangle = |U\rangle\rangle * |U_C\rangle\rangle$ (cf. Eq. (9)), which, with the decomposition in Eq. (24), can be written as $|U_1\rangle\rangle * |\mathbb{1}\rangle\rangle^{Z\bar{Z}} * |U_2\rangle\rangle * |U_C\rangle\rangle$. Therefore, with Eq. (13) of the main text, the term in the round bracket on the right-hand side of Eq. (31) is precisely $|U_C\rangle\rangle * |\mathbb{1}\rangle\rangle^{Z\bar{Z}}$. Finally, evaluating the link product over Z and $\bar{Z}$ yields

$$\begin{aligned} |R(U_C)\rangle\rangle * |R'\rangle\rangle &= |U_1\rangle\rangle^{P_O A_O B_O C_I Z} * (|U_C\rangle\rangle^{C_{IO} C'_{IO}} \otimes |\mathbb{1}\rangle\rangle^{Z\bar{Z}}) * |U_2\rangle\rangle^{C_O \bar{Z} A_I B_I F_I} \\ &= (|U_1\rangle\rangle^{P_O A_O B_O C_I Z} * |\mathbb{1}\rangle\rangle^{Z\bar{Z}} * |U_2\rangle\rangle^{C_O \bar{Z} A_I B_I F_I}) * |U_C\rangle\rangle^{C_{IO} C'_{IO}} = |U\rangle\rangle^{P_O A_{IO} B_{IO} C_{IO} F} * |U_C\rangle\rangle^{C_{IO} C'_{IO}} . \end{aligned} \tag{32}$$

(see Fig. 4(c)).

SUPPLEMENTARY NOTE 4—EXAMPLE OF A PROCESS THAT VIOLATES CAUSAL INEQUALITIES ON TIME-DELOCALISED SUBSYSTEMS

A. Description in terms of quantum operations and time-delocalised subsystems

The first operation in the circuit of Fig. 5, $\omega_1(U_C) : \mathcal{H}^{P_1P_2P_3C'_I \to \bar{T}_1E_1\bar{Q}_1\gamma}$ is given by

$$\omega_1(U_C) = \mathbb{1}^{P_1\to\bar{T}_1} \otimes \mathbb{1}^{P_2\to E_1} \otimes [(|0\rangle^{\bar{Q}_1} \langle 0|^{C_O} \otimes \mathbb{1}^{C'_O\to\gamma}) \cdot U_C \cdot (\mathbb{1}^{P_3\to C_I} \otimes \mathbb{1}^{C'_I})] + \mathbb{1}^{P_1\to E_1} \otimes \mathbb{1}^{P_2\to\bar{T}_1} \otimes [(|1\rangle^{\bar{Q}_1} \langle 1|^{C_O} \otimes \mathbb{1}^{C'_O\to\gamma}) \cdot U_C \cdot (\mathbb{1}^{P_3\to C_I} \otimes \mathbb{1}^{C'_I})], \quad (33)$$

where γ is an ancillary space with dimension $d_\gamma = d_{C'_I}$. The two circuit operations $\omega_2^\circ : \mathcal{H}^{\bar{T}'_1E_1} \to \mathcal{H}^{\bar{T}_2E_2}$ and $\omega_2^\bullet : \mathcal{H}^{\bar{T}'_1E_1} \to \mathcal{H}^{\bar{T}_2E_2}$ do not depend on U_C for this particular process. They are

$$\omega_2^\circ = |0\rangle^{E_2} \langle 0|^{\bar{T}'_1} \otimes \mathbb{1}^{E_1\to\bar{T}_2} + |1\rangle^{E_2} \langle 1|^{\bar{T}'_1} \otimes \sigma_{\mathrm{x}}^{E_1\to\bar{T}_2} \quad (34)$$

(where $\sigma_{\mathrm{x}}^{Y\to Z} := |0\rangle^Z \langle 1|^Y + |1\rangle^Z \langle 0|^Y$ denotes a NOT gate) and

$$\omega_2^\bullet = |1\rangle^{E_2} \langle 1|^{\bar{T}'_1} \otimes \mathbb{1}^{E_1\to\bar{T}_2} + |0\rangle^{E_2} \langle 0|^{\bar{T}'_1} \otimes \sigma_{\mathrm{x}}^{E_1\to\bar{T}_2}. \quad (35)$$

The final circuit operation $\omega_3(U_C) : \mathcal{H}^{\bar{T}'_2E_2\bar{Q}'_2\gamma} \to \mathcal{H}^{F_1F_2F_3C'_O}$ is

$$\begin{aligned}\omega_3(U_C) = \big[\, &|000\rangle^{F_1F_2F_3} \langle 000|^{\bar{T}'_2E_2\bar{Q}'_2} + |001\rangle^{F_1F_2F_3} \langle 001|^{\bar{T}'_2E_2\bar{Q}'_2} + |100\rangle^{F_1F_2F_3} \langle 010|^{\bar{T}'_2E_2\bar{Q}'_2} \\ &+ |101\rangle^{F_1F_2F_3} \langle 101|^{\bar{T}'_2E_2\bar{Q}'_2} + |110\rangle^{F_1F_2F_3} \langle 110|^{\bar{T}'_2E_2\bar{Q}'_2} + |111\rangle^{F_1F_2F_3} \langle 111|^{\bar{T}'_2E_2\bar{Q}'_2} \big] \otimes \mathbb{1}^{\gamma\to C'_O} \\ &+ |01\rangle^{F_1F_2} \langle 100|^{\bar{T}'_2E_2\bar{Q}'_2} \otimes \big[\mathbb{1}^{C_O\to F_3} \otimes \mathbb{1}^{C'_O}) \cdot (U_C \cdot (\sigma_{\mathrm{x}}^{C_I\to C_I} \otimes \mathbb{1}^{C'_I}) \cdot U_C^\dagger) \cdot (|0\rangle^{C_O} \otimes \mathbb{1}^{\gamma\to C'_O}\big] \\ &+ |01\rangle^{F_1F_2} \langle 011|^{\bar{T}'_2E_2\bar{Q}'_2} \otimes \big[\mathbb{1}^{C_O\to F_3} \otimes \mathbb{1}^{C'_O}) \cdot (U_C \cdot (\sigma_{\mathrm{x}}^{C_I\to C_I} \otimes \mathbb{1}^{C'_I}) \cdot U_C^\dagger) \cdot (|1\rangle^{C_O} \otimes \mathbb{1}^{\gamma\to C'_O}\big]. \end{aligned} \quad (36)$$

For this particular process, a decomposition as in Eq. (24) is given by $|U_{\mathrm{BW}}\rangle\rangle = |U_1\rangle\rangle * |\mathbb{1}\rangle\rangle^{Z\bar{Z}} * |U_2\rangle\rangle$, with the unitaries $U_1 : \mathcal{H}^{P_1P_2P_3A_OB_O} \to \mathcal{H}^{C_IZ}$ and $U_2 : \mathcal{H}^{C_O\bar{Z}} \to \mathcal{H}^{A_IB_IF_1F_2F_3}$

$$\begin{aligned} U_1 &= \sum_{p_1p_2p_3a_Ob_O} |p_3 \oplus \neg\, a_O \wedge b_O\rangle^{C_I} |p_1,p_2,a_O,b_O\rangle^Z \langle p_1,p_2,p_3|^{P_1P_2P_3} \langle a_O,b_O|^{A_OB_O} \\ U_2 &= \sum_{a_Ob_Oc_Op_1p_2} |p_1 \oplus \neg\, b_O \wedge c_O, p_2 \oplus \neg\, c_O \wedge a_O\rangle^{A_IB_I} |a_O,b_O,c_O\rangle^{F_1F_2F_3} \langle c_O|^{C_O} \langle p_1,p_2,a_O,b_O|^{\bar{Z}}, \end{aligned} \quad (37)$$

from which the isomorphisms $J_{\mathrm{in}} : \mathcal{H}^{A_IB_IC_IYZ} \to \mathcal{H}^{T_1T_2\bar{T}'_1\bar{T}'_2Q_1P_O}$ and $J_{\mathrm{out}} : \mathcal{H}^{T'_1T'_2\bar{T}_1\bar{T}_2Q'_2F_I} \to \mathcal{H}^{A_OB_OC_O\bar{Y}\bar{Z}}$ that define the decomposition into time-delocalised subsystems are obtained through Eqs. (25) and (26).

Specifically, these are given by

$$\begin{aligned} J_{\mathrm{in}} =& \mathbb{1}^{A_I\to T_1} \otimes \mathbb{1}^{B_I\to T_2} \otimes [\sum_{\substack{p_1p_2p_3\\a_Ob_O}} |p_1,p_2,p_3\rangle^{P_1P_2P_3} |a_O,b_O\rangle^{\bar{T}'_1\bar{T}'_2} \langle p_3 \oplus \neg\, a_O \wedge b_O|^{C_I} \langle p_1,p_2,a_O,b_O|^Z] \otimes |0\rangle^{Q_1} \langle 0|^Y \\ &+ \mathbb{1}^{B_I\to T_1} \otimes \mathbb{1}^{A_I\to T_2} \otimes [\sum_{\substack{p_1p_2p_3\\a_Ob_O}} |p_1,p_2,p_3\rangle^{P_1P_2P_3} |a_O,b_O\rangle^{\bar{T}'_2\bar{T}'_1} \langle p_3 \oplus \neg\, a_O \wedge b_O|^{C_I} \langle p_1,p_2,a_O,b_O|^Z] \otimes |1\rangle^{Q_1} \langle 1|^Y, \end{aligned} \quad (38)$$

and

$$\begin{aligned} J_{\mathrm{out}} =& \mathbb{1}^{T'_1\to A_O} \otimes \mathbb{1}^{T'_2\to B_O} \\ &\otimes [\sum_{\substack{a_Ob_Oc_O\\p_1p_2}} |c_O\rangle^{C_O} |p_1,p_2,a_O,b_O\rangle^{\bar{Z}} \langle p_1 \oplus \neg\, b_O \wedge c_O, p_2 \oplus \neg\, c_O \wedge a_O|^{\bar{T}_1\bar{T}_2} \langle a_O,b_O,c_O|^{F_1F_2F_3}] \otimes |0\rangle^{\bar{Y}} \langle 0|^{Q'_2} \\ &+ \mathbb{1}^{T'_1\to B_O} \otimes \mathbb{1}^{T'_2\to A_O} \\ &\otimes [\sum_{\substack{a_Ob_Oc_O\\p_1p_2}} |c_O\rangle^{C_O} |p_1,p_2,a_O,b_O\rangle^{\bar{Z}} \langle p_1 \oplus \neg\, b_O \wedge c_O, p_2 \oplus \neg\, c_O \wedge a_O|^{\bar{T}_2\bar{T}_1} \langle a_O,b_O,c_O|^{F_1F_2F_3}] \otimes |1\rangle^{\bar{Y}} \langle 1|^{Q'_2}. \end{aligned} \quad (39)$$

These isomorphisms are represented graphically in Supplementary Fig. 10.

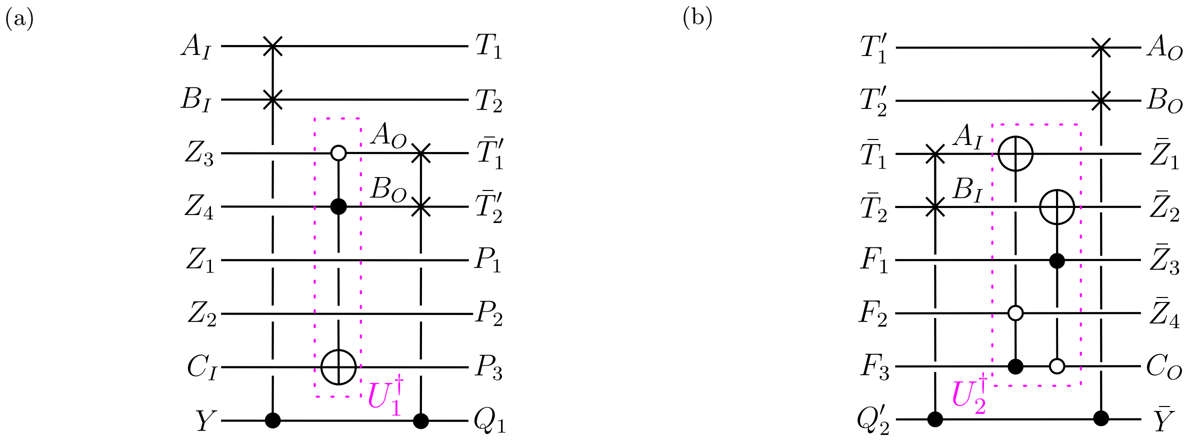

Supplementary Fig. 10. Graphical representation of the isomorphisms $J_{\rm in}$ and $J_{\rm out}$ for the BW process. (a) Isomorphism $J_{\rm in}$ that defines the time-delocalised subsystems A_I, B_I, and C_I for the particular example of the BW process from Eq. (5) and Fig. 5 (with the four qubits constituting $\mathcal{H}^Z := \mathcal{H}^{Z_1} \otimes \mathcal{H}^{Z_2} \otimes \mathcal{H}^{Z_3} \otimes \mathcal{H}^{Z_4}$ shown as separate systems).
(b) Isomorphism $J_{\rm out}$ that defines the time-delocalised subsystems A_O, B_O, and C_O (with again the four qubits constituting $\mathcal{H}^{\bar{Z}} := \mathcal{H}^{\bar{Z}_1} \otimes \mathcal{H}^{\bar{Z}_2} \otimes \mathcal{H}^{\bar{Z}_3} \otimes \mathcal{H}^{\bar{Z}_4}$ shown as separate systems).

One can check that by applying the general tripartite "circuit rewriting" procedure (represented graphically in Fig. 4 and detailled mathematically in Supplementary Note 3 C) to the particular temporal circuit of Fig. 5, with the specific circuit operations ω_1, ω_2°, $\omega_2^\bullet$, ω_3 in Eqs. (33)–(36) and the specific isomorphisms $J_{\rm in}$ and $J_{\rm out}$ defined in Eqs. (38) and (39), one indeed ends up with the process vector $|U_{\rm BW}\rangle\rangle$ in Eq. (5) of the main text, composed with $|U_A\rangle\rangle$, $|U_B\rangle\rangle$ and $|U_C\rangle\rangle$. (And where, in the calculation, one replaces $|\omega_1^{[0]}(U_C)\rangle\rangle^{P_O C_I' \bar{T}_1 E_1 \bar{Q}_1}$ by $|\omega_1(U_C)\rangle\rangle^{P_1 P_2 P_3 C_I' \bar{T}_1 E_1 \bar{Q}_1 \gamma}$, and $|\omega_3^{[0]}(U_C)\rangle\rangle^{\bar{T}_2' E_2 \bar{Q}_2' F_I C_O'}$ by $|\omega_1(U_C)\rangle\rangle^{\bar{T}_2' E_2 \bar{Q}_2' \gamma F_1 F_2 F_3 C_O'}$, in order to account for the simplifications we made in the treatment of the circuit ancillas, see Fig. 5). The operations $R(U_C)$ and R' for this specific example, as well as their composition, are shown in Supplementary Figs. 11 to 14.

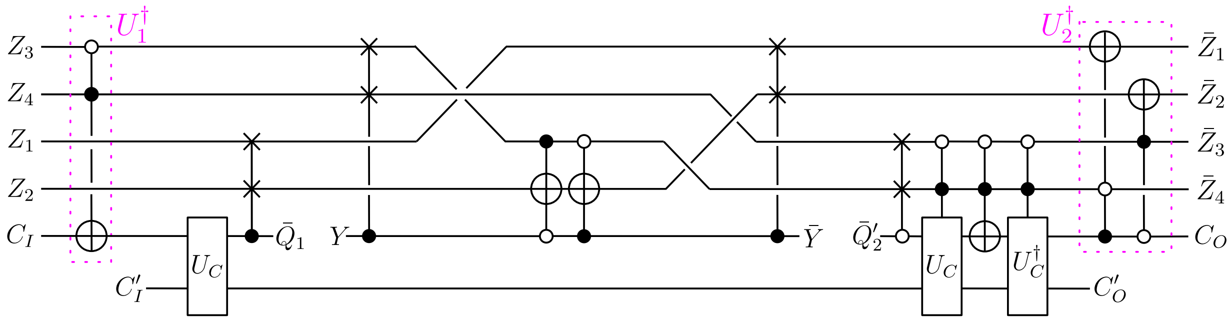

Supplementary Fig. 11. Circuit representation of $R(U_C)$ for the BW process.

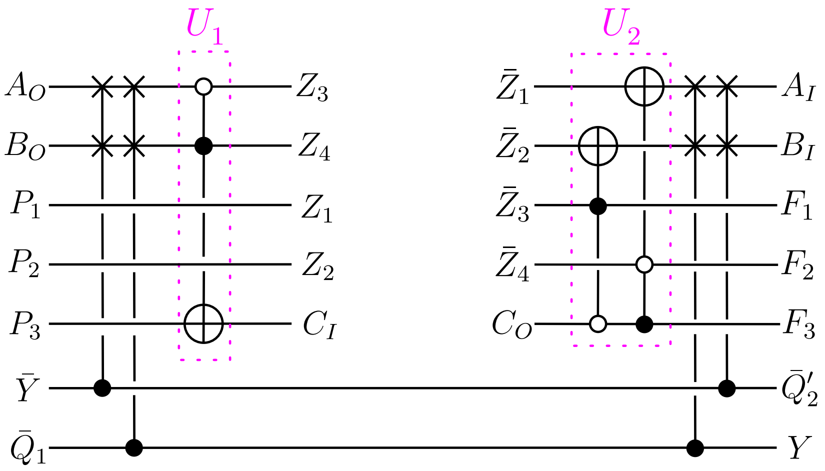

Supplementary Fig. 12. Circuit representation of R' for the BW process.

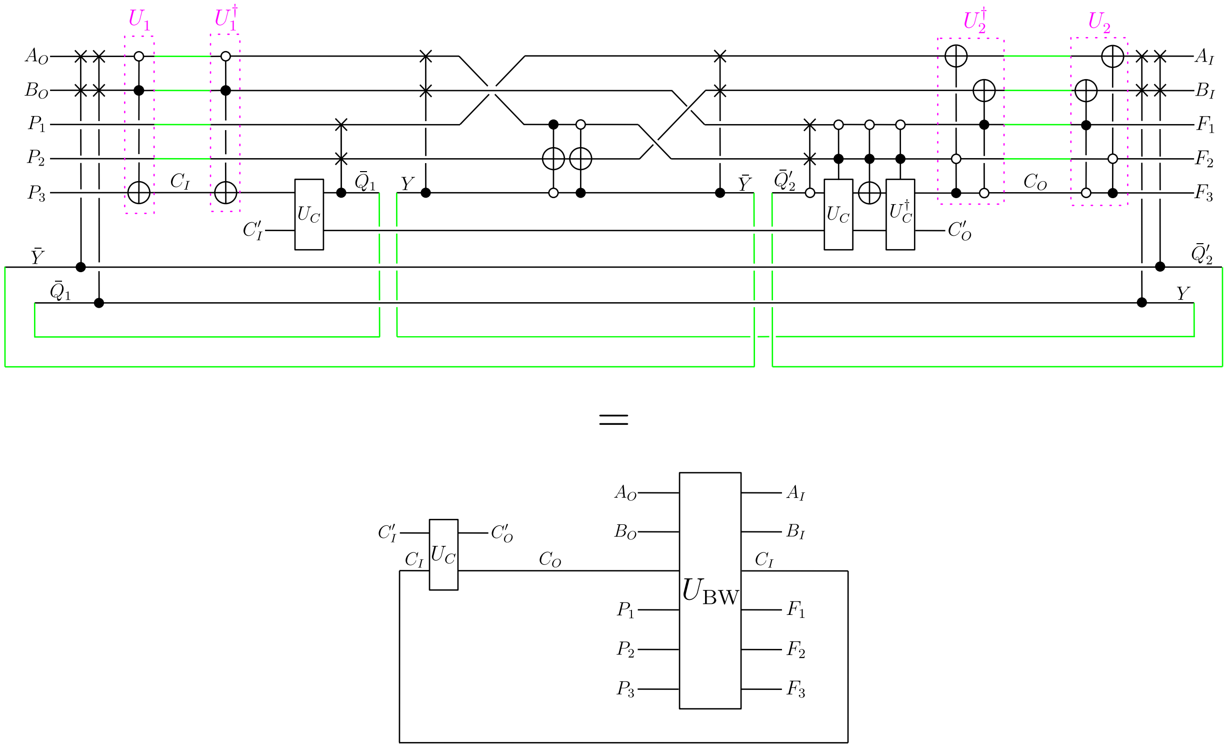

Supplementary Fig. 13. Circuit representation of the composition of $R(U_C)$ and R' for the BW process. Here we see in particular how the time-delocalised systems (or classical variables, here) C_I, C_O are identified. To verify that we indeed get the composition of U_{BW} with U_C, we should evaluate the composition over C_I, C_O, as we do on the next figure.

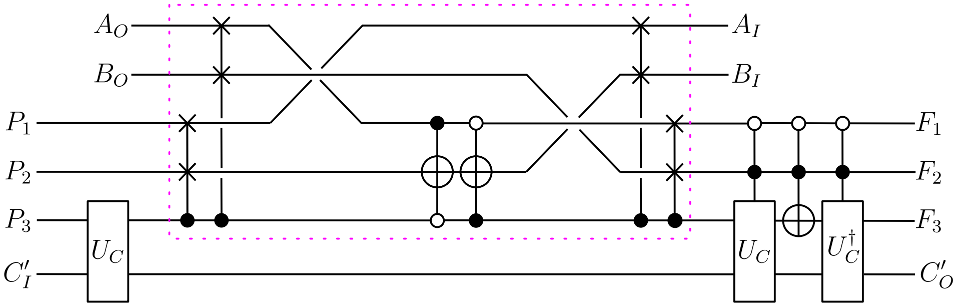

Supplementary Fig. 14. Circuit representation of the composition of $R(U_C)$ and R' for the BW process, after simplification of $U_1^\dagger U_1$ and $U_2 U_2^\dagger$ and other further simplifications in the previous figure. It is easily checked that the circuit fragment in the dashed box realises the unitary operation $\sum_{a_O,b_O,c_O,p_1,p_2} |p_1 \oplus \neg b_O \wedge c_O, p_2 \oplus \neg c_O \wedge a_O, a_O, b_O, c_O\rangle\langle a_O, b_O, p_1, p_2, c_O|$, so that the whole fragment shown above realises $\sum_{a_O,b_O,c_O,p_1,p_2} |p_1 \oplus \neg b_O \wedge c_O, p_2 \oplus \neg c_O \wedge a_O, a_O, b_O\rangle\langle a_O, b_O, p_1, p_2| \otimes [U_C(\sigma_{\rm x} \otimes \mathbb{1})U_C^\dagger]^{\neg a_O \wedge b_O}(|c_O\rangle\langle c_O| \otimes \mathbb{1})U_C = \sum_{a_O,b_O,c_O,p_1,p_2,p_3} |p_1 \oplus \neg b_O \wedge c_O, p_2 \oplus \neg c_O \wedge a_O, a_O, b_O\rangle\langle a_O, b_O, p_1, p_2| \otimes (|c_O\rangle\langle c_O| \otimes \mathbb{1})U_C(|p_3 \oplus \neg a_O \wedge b_O\rangle\langle p_3| \otimes \mathbb{1})$ (where the equality is obtained rather trivially for the terms with $\neg a_O \wedge b_O = 0$; for the terms with $\neg a_O \wedge b_O = 1$, note that c_O only appears in $\sum_{c_O} |c_O\rangle\langle c_O| = \mathbb{1}$; we can then simplify this partial sum, simplify $U_C^\dagger$ together with U_C, re-introduce a similar $\sum_{c_O} |c_O\rangle\langle c_O|$ to the left of the remaining U_C, and finally write $\sigma_{\rm x}^{\neg a_O \wedge b_O} = \sum_{p_3} |p_3 \oplus \neg a_O \wedge b_O\rangle\langle p_3|$). From this expression it is then easy to verify that the above circuit indeed realises precisely the composition of the process $U_{\rm BW}$, as defined from Eq. (5) in the main text, with U_C (see Fig. 4(c)).

B. Description in terms of classical operations and time-delocalised variables

In this Supplementary Note, we explain in more detail that the example in "A process that violates causal inequalities on time-delocalised subsystems" of the main text describes a *classical* noncausal process, with classical operations that take place on *time-delocalised variables*. The classical counterpart of a quantum system X with a Hilbert space $\mathcal{H}^X$ is a random variable X with values in a set $\mathcal{S}^X := \{0, \ldots, d_X - 1\}$. The classical counterpart of a quantum operation (i.e., most generally, of a quantum instrument) from an incoming quantum system X to an outgoing quantum system Y is a "classical instrument", that is, a conditional probability distribution $P(r, y|x)$ which specifies the probability that the outgoing variable Y takes the value $y \in \mathcal{S}^Y$ and the "outcome" of the operation (which can also be described by a random variable) is r, given that the incoming variable X has the value $x \in \mathcal{S}^X$.

If the outgoing variable Y is then passed on to a subsequent classical operation (say, an operation from incoming random variables S and Y to outgoing random variables T and Z, specified by a conditional probability distribution $P(t, z|s, y)$), the two operations compose to a new classical operation $P(r, t, z|x, s) = \sum_y P(r, y|x)P(t, z|s, y)$. This is the classical counterpart of the link product.

If one then has an acyclic network composed of classical operations, it can be described in terms of *time-delocalised variables* by decomposing it into fragments and composing these with deterministic reversible operations that take the incoming and outgoing "time-local" variables to new ones, analogously to the quantum case. For instance, if the operations in the circuit of Fig. 2 are all classical, the red fragment defines a classical operation $P_{\rm red}(k, d, h, i|a, f)$ from incoming time-local variables A, F to outgoing time-local variables D, H, I and with an outcome k, and the blue circuit fragment an operation $P_{\rm blue}(j, l, m, a, f|d, h, i)$, from the incoming variables D, H, I to the outgoing variables A, F and with outcomes j, l, m. We can then change to a description in terms of time-delocalised variables V, W, X, Y defined by any bijective functions $J_{\rm in} : \mathcal{S}^V \times \mathcal{S}^W \to \mathcal{S}^A \times \mathcal{S}^F$ and $J_{\rm out} : \mathcal{S}^D \times \mathcal{S}^H \times \mathcal{S}^I \to \mathcal{S}^X \times \mathcal{S}^Y$, by taking

$$P'_{\rm red}(k, x, y|v, w) = \sum_{a,d,f,h,i} P_{\rm red}(k, d, h, i|a, f)\delta_{(a,f),J_{\rm in}(v,w)}\delta_{(x,y),J_{\rm out}(d,h,i)} \tag{40}$$

and

$$P'_{\rm blue}(j, l, m, v, w|x, y) = \sum_{a,d,f,h,i} P_{\rm blue}(j, l, m, a, f|d, h, i)\delta_{(d,h,i),J_{\rm out}^{-1}(x,y)}\delta_{(v,w),J_{\rm in}^{-1}(a,f)}. \tag{41}$$

where $\delta_{(\ldots),(\ldots)}$ denotes the Kronecker delta between tuples. It is straightforward to check that, analogously to the quantum case, the composition of the two fragments over the time-local and time-delocalised variables is indeed the same—i.e., that $\sum_{v,w,x,y} P'_{\rm red}(k, x, y|v, w)P'_{\rm blue}(j, l, m, v, w|x, y) = \sum_{a,d,f,h,i} P_{\rm red}(k, d, h, i|a, f)P_{\rm blue}(j, l, m, a, f|d, h, i) = P(j, k, l, m)$.

If the state evolving through a quantum circuit is diagonal in the computational basis at any (relevant) time, the situation is effectively classical (i.e., when one identifies each quantum system X with a classical, random variable X and the computational basis states $|i\rangle^X$ of $\mathcal{H}^X$ with the elements of $\mathcal{S}^X$, one obtains a probability distribution evolving through a circuit consisting of classical operations in the above sense). In the circuit of Fig. 5 for instance, when an input state in the incoming systems $\mathcal{H}^{P_1P_2P_3A'_IB'_IC'_I}$ that is diagonal in the computational basis is prepared, and when U_A, U_B and U_C are restricted to unitaries that map computational basis states to computational basis states, we indeed have such an effectively classical circuit composed of deterministic operations.

The quantum isomorphisms $J_{\text{in}} : \mathcal{H}^{A_IB_IC_IYZ} \to \mathcal{H}^{T_1T_2\bar{T}'_1\bar{T}'_2Q_1P_O}$ and $J_{\text{out}} : \mathcal{H}^{T'_1T'_2\bar{T}_1\bar{T}_2Q'_2F_I} \to \mathcal{H}^{A_OB_OC_O\bar{Y}\bar{Z}}$ given in Supplementary Note 4 A above, which also map computational basis states to computational basis states, then translate into bijective functions $J_{\text{in}} : \mathcal{S}^{A_I} \times \mathcal{S}^{B_I} \times \mathcal{S}^{C_I} \times \mathcal{S}^Y \times \mathcal{S}^Z \to \mathcal{S}^{T_1} \times \mathcal{S}^{T_2} \times \mathcal{S}^{\bar{T}'_1} \times \mathcal{S}^{\bar{T}'_2} \times \mathcal{S}^{Q_1} \times \mathcal{S}^{P_O}$ and $J_{\text{out}} : \mathcal{S}^{T'_1} \times \mathcal{S}^{T'_2} \times \mathcal{S}^{\bar{T}_1} \times \mathcal{S}^{\bar{T}_2} \times \mathcal{S}^{Q'_2} \times \mathcal{S}^{F_I} \to \mathcal{S}^{A_O} \times \mathcal{S}^{B_O} \times \mathcal{S}^{C_O} \times \mathcal{S}^{\bar{Y}} \times \mathcal{S}^{\bar{Z}}$ that define the time-delocalised variables A_I, B_I, C_I, A_O, B_O, C_O. Namely, we obtain $A_I = (\neg Q_1 \wedge T_1) \oplus (Q_1 \wedge T_2)$, $B_I = (\neg Q_1 \wedge T_2) \oplus (Q_1 \wedge T_1)$, $C_I = P_3 \oplus (\neg Q_1 \wedge \neg \bar{T}'_1 \wedge \bar{T}'_2) \oplus (Q_1 \wedge \bar{T}'_1 \wedge \neg \bar{T}'_2)$, $A_O = (\neg Q'_2 \wedge T'_1) \oplus (Q'_2 \wedge T'_2)$, $B_O = (\neg Q'_2 \wedge T'_2) \oplus (Q'_2 \wedge T'_1)$ and $C_O = F_3$ (see Supplementary Fig. 10). With respect to these variables, the circuit of Fig. 5 then corresponds to three classical local operations $P_A(a_O, a'_O|a_I, a'_I)$, $P_B(b_O, b'_O|b_I, b'_I)$, $P_C(c_O, c'_O|c_I, c'_I)$, that are composed with a classical, deterministic channel $P_{\text{BW}}(a_I, b_I, c_I, f_1, f_2, f_3|a_O, b_O, c_O, p_1, p_2, p_3) = \delta_{a_I, p_1 \oplus \neg b_O \wedge c_O} \delta_{b_I, p_2 \oplus \neg c_O \wedge a_O} \delta_{c_I, p_3 \oplus \neg a_O \wedge b_O} \delta_{f_1, a_O} \delta_{f_2, b_O} \delta_{f_3, c_O}$ that sends the outputs A_O, B_O, C_O of the local operations, as well as the outputs P_1, P_2 and P_3 of the "global past" party, back to their inputs A_I, B_I, C_I and the inputs F_1, F_2 and F_3 of the "global future" party. When the classical input state $\delta_{0,p_1}\delta_{0,p_2}\delta_{0,p_3}$ is prepared by the "global past" party, and the input variables of the "global future" party are discarded, the operation realised by the circuit can be written as

$$P(a'_O, b'_O, c'_O|a'_I, b'_I, c'_I) = \sum_{\substack{a_I, b_I, c_I, \\ a_O, b_O, c_O}} P_{\text{AF}}(a_I, b_I, c_I|a_O, b_O, c_O) P_A(a_O, a'_O|a_I, a'_I) P_B(b_O, b'_O|b_I, b'_I) P_C(c_O, c'_O|c_I, c'_I), \quad (42)$$

i.e., the circuit corresponds to $P_A(a_O, a'_O|a_I, a'_I)$, $P_B(b_O, b'_O|b_I, b'_I)$, $P_C(c_O, c'_O|c_I, c'_I)$, composed with a deterministic, classical channel $P_{\text{AF}}(a_I, b_I, c_I|a_O, b_O, c_O) = \delta_{a_I, \neg b_O \wedge c_O} \delta_{b_I, \neg c_O \wedge a_O} \delta_{c_I, \neg a_O \wedge b_O}$ (which indeed corresponds to the "classical process matrix" W_{AF} of Eq. (4) in the main text). In this classical description, for the operations that the parties apply to violate causal inequalities (see "A process that violates causal inequalities on time-delocalised subsystems" in the main text), we may identify the incoming ancillary variables A'_I, B'_I and C'_I with the classical variables that describe the local classical input variables, or "settings", that the parties receive (denoted by I_A, I_B and I_C, respectively, with values i_A, i_B and i_C), and their outgoing ancillary variables A'_O, B'_O and C'_O with their classical outputs (described by classical variables O_A, O_B and O_C, respectively, with values o_A, o_B and o_C). The operations are then given by $P(a_O, o_A|a_I, i_A) = \delta_{o_A, a_I} \delta_{a_O, i_A}$, $P(b_O, o_B|b_I, i_B) = \delta_{o_B, b_I} \delta_{b_O, i_B}$ and $P(c_O, o_C|c_I, i_C) = \delta_{o_C, c_I} \delta_{c_O, i_C}$, and through Eq. (42) we obtain the correlation $P(o_A, o_B, o_C|i_A, i_B, i_C) = \delta_{o_A, \neg i_B \wedge i_C} \delta_{o_B, \neg i_C \wedge i_A} \delta_{o_C, \neg i_A \wedge i_B}$, which violates causal inequalities.

SUPPLEMENTARY NOTE 5—CAUSAL INEQUALITY ASSUMPTIONS IMPLY CAUSAL CORRELATIONS

In this section, we are going to prove that the conditions (15)–(18) from the section "Causal inequality assumptions" in the main text imply that $P(o_A, o_B, o_C|i_A, i_B, i_C)$ must respect causal inequalities. For most generality, we consider the general multipartite case, that is, we consider a set of variables $\Gamma := \{X_I, X_O, I_X, O_X\}_{X=A,B,C,...}$ for an arbitrary number of parties $\{A, B, C, \ldots\}$, and a probability distribution $P(o_A, o_B, o_C, \ldots, \kappa(\Gamma)|i_A, i_B, i_C, \ldots)$, with $\kappa(\Gamma)$ the possible SPOs on the set Γ, that satisfies the straightforward multipartite generalisation of the constraints (15)–(18). As in the main text, when we write probabilities for some constraint on the causal order, this is to be understood as the probability obtained by summing over all $\kappa(\Gamma)$ that satisfy the respective constraint. (For instance, $P(I_{\Lambda^{(0)}} \prec \Lambda_I^{(0)}|i_A, i_B, i_C, \ldots)$ below is obtained by summing $P(o_A, o_B, o_C, \ldots, \kappa(\Gamma)|i_A, i_B, i_C, \ldots)$ over all $\kappa(\Gamma)$ that satisfy $I_{\Lambda^{(0)}} \prec \Lambda_I^{(0)}$, as well as over all outcomes.)

The idea of the proof is that, to each $\kappa(\Gamma)$, we associate a "coarse-grained" SPO $\mathcal{C}_{\kappa(\Gamma)}$ on the set of parties $\{A, B, C, \ldots\}$, and show that, with respect to this coarse-grained SPO, the correlation $P(o_A, o_B, o_C, \ldots|i_A, i_B, i_C, \ldots)$ satisfies the definition of "causal correlation" as introduced in Ref. [9] (also referred to as "causal process" in Ref. [9]), which means that it must respect causal inequalities. Let us first recall this definition.

Causal correlation (Definition from Ref. [9]). *A correlation $P(o_A, o_B, o_C, \ldots|i_A, i_B, i_C, \ldots)$ for a set of "local experiments" $\{A, B, C, \ldots\} =: \Delta$, is* causal *if and only if there exists a probability distribution*

$$P(o_A, o_B, o_C, \ldots, \xi(\Delta)|i_A, i_B, i_C, \ldots), \tag{43}$$

where $\xi(\Delta)$ are the possible SPOs on the set Δ, such that

$$\sum_{\xi(\Delta)} P(o_A, o_B, o_C, \ldots, \xi(\Delta)|i_A, i_B, i_C, \ldots) = P(o_A, o_B, o_C, \ldots|i_A, i_B, i_C, \ldots) \tag{44}$$

and such that, for every local experiment (e.g., A), for every subset $\mathcal{X}$ of the rest of the local experiments, and for every SPO $\xi(\{A\} \cup \mathcal{X})$ on the local experiment in question and that subset,

$$P(o^{\mathcal{X}}, A \not\preceq \mathcal{X}, \xi(\{A\} \cup \mathcal{X})|i_A, i_B, i_C, \ldots) = P(o^{\mathcal{X}}, A \not\preceq \mathcal{X}, \xi(\{A\} \cup \mathcal{X})|i_B, i_C, \ldots). \tag{45}$$

That is, in Eq. (45), one considers the probability for all local experiments in $\mathcal{X}$ to be outside of the causal future of A, to display specific outcomes $o^{\mathcal{X}}$ and to have a specific causal order $\xi(\{A\} \cup \mathcal{X})$ together with A. This probability is required to be independent of the setting of the local experiment A.

We thus consider the coarse-grained set $\{A, B, C, \ldots\} = \Delta$, and first define a binary relation $\mathcal{C}_{\kappa(\Gamma)}$ on this set, for any given $\kappa(\Gamma)$, as follows: $X\mathcal{C}_{\kappa(\Gamma)}Y$ if and only if there exist some parties $\Lambda^{(1)}, \Lambda^{(2)}, \ldots, \Lambda^{(M)} \in \Delta$, $M \geq 0$, such that $X_O \prec \Lambda_I^{(1)}$, $\Lambda_O^{(i)} \prec \Lambda_I^{(i+1)}$ for $1 \leq i \leq M-1$, and $\Lambda_O^{(M)} \prec Y_I$. (We also allow for $M = 0$, which corresponds to the case where $X_O \prec Y_I$, without any "intermediate" Λ.)

As a first step, we show that, for all $\kappa(\Gamma)$ that occur with non-zero probability, this coarse-grained relation $\mathcal{C}_{\kappa(\Gamma)}$ is indeed a SPO on the set $\{A, B, C, \ldots\}$. That is, we prove the following.

Proposition 1. *$P(o_A, o_B, o_C, \ldots, \kappa(\Gamma)|i_A, i_B, i_C, \ldots) > 0$ only if $\mathcal{C}_{\kappa(\Gamma)}$ is a SPO on the set of coarse-grained variables Δ.*

Proof. It is straightforward to check that $\mathcal{C}_{\kappa(\Gamma)}$ is transitive. We thus need to show its irreflexivity for all $\kappa(\Gamma)$ associated with a non-zero probability. $\mathcal{C}_{\kappa(\Gamma)}$ is reflexive if and only if there exist $\Lambda^{(0)}, \Lambda^{(1)}, \ldots, \Lambda^{(M)} \in \Delta$, $M \geq 0$, such that $\Lambda_O^{(i)} \prec \Lambda_I^{(i+1)}$ for $0 \leq i \leq M-1$ and $\Lambda_O^{(M)} \prec \Lambda_I^{(0)}$. We prove by induction over M that, for any $\kappa(\Gamma)$ for which such $\Lambda^{(0)}, \Lambda^{(1)}, \ldots, \Lambda^{(M)}$ exist, the probabilities $P(o_A, o_B, o_C, \ldots, \kappa(\Gamma)|i_A, i_B, i_C, \ldots)$ must be zero.

We start by proving the claim for $M = 0$. Assume thus that, for the $\kappa(\Gamma)$ under consideration, we have $\Lambda_O^{(0)} \prec \Lambda_I^{(0)}$. Since $I_{\Lambda^{(0)}} \prec \Lambda_O^{(0)}$ (as per condition (17) of the closed laboratory assumption in the main text), transitivity implies that, for this $\kappa(\Gamma)$, we have $I_{\Lambda^{(0)}} \prec \Lambda_I^{(0)}$. However, the probability $P(I_{\Lambda^{(0)}} \prec \Lambda_I^{(0)}|i_A, i_B, i_C, \ldots)$ (and therefore any probability $P(o_A, o_B, o_C, \ldots, \kappa(\Gamma)|i_A, i_B, i_C, \ldots)$ with $\kappa(\Gamma)$ satisfying $I_{\Lambda^{(0)}} \prec \Lambda_I^{(0)}$ that contributes to it) must be zero. This follows from the fact that $P(I_{\Lambda^{(0)}} \prec \Lambda_I^{(0)}|i_A, i_B, i_C, \ldots)$ is independent of $i_{\Lambda^{(0)}}$ (since it is obtained by summing over various probabilities that are each individually independent of $i_{\Lambda^{(0)}}$ according to Eq. (16) in the main text; namely, to obtain $P(I_{\Lambda^{(0)}} \prec \Lambda_I^{(0)}|i_A, i_B, i_C, \ldots)$ one sums the constraint of Eq. (16) corresponding to $I_{\Lambda^{(0)}}$ over all $\mathcal{Y}, \mathcal{Z}$ such that $\Lambda_I^{(0)} \notin \mathcal{Y} \cup \mathcal{Z}$, all causal orders on the respective $\mathcal{Y} \cup \mathcal{Z}$, and all outcomes in the respective $\mathcal{Y} \cup \mathcal{Z}$),

and $P(I_{\Lambda^{(0)}} \prec \Lambda_I^{(0)}|i_A, i_B, i_C, \ldots) = 0$ for the value $i^*_{\Lambda^{(0)}}$ of $I_{\Lambda^{(0)}}$ (since, for $i^*_{\Lambda^{(0)}}$, we have $\Lambda_I^{(0)} \prec \Lambda_O^{(0)}$ with certainty, which is incompatible with $I_{\Lambda^{(0)}} \prec \Lambda_I^{(0)}$, as the latter would imply $\Lambda_O^{(0)} \prec \Lambda_I^{(0)}$ due to condition (17) in the main text).

Assume then that the claim holds up to some given $M \geq 0$—i.e., that whenever $\kappa(\Gamma)$ is such that some $\Lambda^{(0)}, \ldots, \Lambda^{(M)}$ with the prescribed properties exist (starting from any element of Δ), then $P(o_A, o_B, o_C, \ldots, \kappa(\Gamma)|i_A, i_B, i_C, \ldots) = 0$. We will prove that the claim then also holds for $M+1$. We thus consider a $\kappa(\Gamma)$ such that there exist $\Lambda^{(0)}, \ldots, \Lambda^{(M+1)}$, for which $\Lambda_O^{(i)} \prec \Lambda_I^{(i+1)}$ for $0 \leq i \leq M$ and $\Lambda_O^{(M+1)} \prec \Lambda_I^{(0)}$. We consider the sets of variables $\{\Lambda_I^{(i)}|1 \leq i \leq M+1\} =: \Omega_I$ and $\{\Lambda_O^{(i)}|0 \leq i \leq M\} =: \Omega_O$, and distinguish between two possible cases. First, consider the case where any of the variables in $\Omega_I \cup \Omega_O$ is in the causal future of $I_{\Lambda^{(M+1)}}$. If $I_{\Lambda^{(M+1)}} \prec \Lambda_I^{(j)}$ for some $\Lambda_I^{(j)} \in \Omega_I$ (i.e. for some j with $1 \leq j \leq M+1$), the closed laboratory assumption implies that $\Lambda_O^{(M+1)} \prec \Lambda_I^{(j)}$. Therefore, we have $\Lambda_O^{(j)} \prec \Lambda_I^{(j+1)}$, $\Lambda_O^{(j+1)} \prec \Lambda_I^{(j+2)}$, ..., $\Lambda_O^{(M+1)} \prec \Lambda_I^{(j)}$, and thus $\Lambda^{(j)}\mathcal{C}_{\kappa(\Gamma)}\Lambda^{(j)}$ with a number of intermediate Λ strictly smaller than $M+1$, which is associated to a probability of zero by assumption. And if $I_{\Lambda^{(M+1)}} \prec \Lambda_O^{(j)}$ for some $\Lambda_O^{(j)} \in \Omega_O$ (i.e. for some j with $0 \leq j \leq M$), we have that $I_{\Lambda^{(M+1)}} \prec \Lambda_I^{(j+1)}$ because of transitivity.

In the second case, all variables in the sets $\Omega_I \cup \Omega_O$ are in the causal past or elsewhere of $I_{\Lambda^{(M+1)}}$. Therefore, $\kappa(\Gamma)$ contributes to the probability

$$P(\Omega_I \cup \Omega_O \subseteq \mathcal{P}_{I_{\Lambda^{(M+1)}}} \cup \mathcal{E}_{I_{\Lambda^{(M+1)}}}, \Lambda_O^{(i)} \prec \Lambda_I^{(i+1)} \text{ for } 0 \leq i \leq M, I_{\Lambda^{(M+1)}} \prec \Lambda_I^{(0)}|i_A, i_B, i_C, \ldots). \tag{46}$$

This probability is again independent of $i_{\Lambda^{(M+1)}}$, and zero for $i^*_{\Lambda^{(M+1)}}$, implying that it (and therefore also the probability for any $\kappa(\Gamma)$ contributing to it) is always zero. Therefore, it follows that $P(o_A, o_B, o_C, \ldots, \kappa(\Gamma)|i_A, i_B, i_C, \ldots)$ is zero also in this case with $M+1$.

To see that the probability in Eq. (46) is indeed independent of $i_{\Lambda^{(M+1)}}$, note that it is again obtained by summing over various probabilities that are independent of $i_{\Lambda^{(M+1)}}$ according to Eq. (16) in the main text. Namely, one considers the constraint of Eq. (16) corresponding to $I_{\Lambda^{(M+1)}}$, and sums it over all $\mathcal{Y}, \mathcal{Z}$ such that $\Omega_I \cup \Omega_O \subseteq \mathcal{Y} \cup \mathcal{Z}$ and $\Lambda_I^{(0)} \notin \mathcal{Y} \cup \mathcal{Z}$, as well as over all causal orders on the respective $\mathcal{Y} \cup \mathcal{Z}$ that satisfy $\Lambda_O^{(i)} \prec \Lambda_I^{(i+1)}$ for $0 \leq i \leq M$, and over all outcomes in the respective $\mathcal{Y} \cup \mathcal{Z}$.

To see that the probability in Eq. (46) is indeed zero for $i^*_{\Lambda^{(M+1)}}$, note that, for $i^*_{\Lambda^{(M+1)}}$, we have $\Lambda_I^{(M+1)} \prec \Lambda_O^{(M+1)}$, and thus, because of $\Lambda_O^{(M)} \prec \Lambda_I^{(M+1)}$, $\Lambda_O^{(M+1)} \prec \Lambda_I^{(0)}$ and transitivity, $\Lambda_O^{(M)} \prec \Lambda_I^{(0)}$. Therefore, we have $\Lambda^{(0)}\mathcal{C}_{\kappa(\Gamma)}\Lambda^{(0)}$ with a number M of intermediate Λ, which by assumption, implies that the probability associated to $\kappa(\Gamma)$ is zero. □

The second step is then to prove that the probability $P(o_A, o_B, o_C, \ldots|i_A, i_B, i_C, \ldots)$ satisfies the definition of *causal correlation* from Ref. [9], which we recalled above.

Proposition 2. *$P(o_A, o_B, o_C, \ldots|i_A, i_B, i_C, \ldots)$ is a causal correlation, with the underlying probability distribution $P(o_A, o_B, o_C, \ldots, \xi(\Delta)|i_A, i_B, i_C, \ldots)$ associated to each SPO $\xi(\Delta)$ obtained by summing over all $\kappa(\Gamma)$ for which $\mathcal{C}_{\kappa(\Gamma)} = \xi(\Delta)$:*

$$P(o_A, o_B, o_C, \ldots, \xi(\Delta)|i_A, i_B, i_C, \ldots) = \sum_{\{\kappa(\Gamma)|\mathcal{C}_{\kappa(\Gamma)}=\xi(\Delta)\}} P(o_A, o_B, o_C, \ldots, \kappa(\Gamma)|i_A, i_B, i_C, \ldots). \tag{47}$$

Proof. It is clear that this distribution satisfies Eq. (44), since summing $P(o_A, o_B, o_C, \ldots, \xi(\Delta)|i_A, i_B, i_C, \ldots)$ over all $\xi(\Delta)$ is equivalent to summing $P(o_A, o_B, o_C, \ldots, \kappa(\Gamma)|i_A, i_B, i_C, \ldots)$ over all $\kappa(\Gamma)$. It therefore remains to prove that this distribution satisfies Eq. (45).

In order to obtain the probability in Eq. (45), for a given A, $\mathcal{X}$ and $\xi(\{A\}\cup\mathcal{X})$, one needs to sum up the probabilities $P(o_A, o_B, o_C, \ldots, \kappa(\Gamma)|i_A, i_B, i_C, \ldots)$ over all o_X with $X \notin \mathcal{X}$, as well as over all "fine-grained" orders $\kappa(\Gamma)$ for which the "coarse-grained" order $\mathcal{C}_{\kappa(\Gamma)}$ satisfies the respective conditions—that is, with respect to $\mathcal{C}_{\kappa(\Gamma)}$, (*i*) $A \nprec \mathcal{X}$, and (*ii*) the restriction of the coarse-grained order to $\{A\} \cup \mathcal{X}$ is precisely $\xi(\{A\} \cup \mathcal{X})$. Expressed as conditions on the fine-grained $\kappa(\Gamma)$, this means that $\kappa(\Gamma)$ has to satisfy the following two constraints.

$$\begin{aligned}
&(i)\ \forall\ X \in \mathcal{X} : (A_O \nprec X_I) \text{ and } (\nexists\ \Lambda^{(1)}, \ldots, \Lambda^{(M)} \text{ s.t. } A_O \prec \Lambda_I^{(1)}, \Lambda_O^{(i)} \prec \Lambda_I^{(i+1)} \text{ for } 1 \leq i \leq M-1, \text{ and } \Lambda_O^{(M)} \prec X_I) \\
&(ii)\ \forall\ X, Y \in \{A\} \cup \mathcal{X} : [X \prec Y \text{ w.r.t } \xi(\{A\} \cup \mathcal{X})] \\
&\qquad \Leftrightarrow [(X_O \prec Y_I) \text{ or } (\exists\ \Lambda^{(1)}, \ldots, \Lambda^{(M)} \text{ s.t. } X_O \prec \Lambda_I^{(1)}, \Lambda_O^{(i)} \prec \Lambda_I^{(i+1)} \text{ for } 1 \leq i \leq M-1, \text{ and } \Lambda_O^{(M)} \prec Y_I)].
\end{aligned} \tag{48}$$

To proceed, we note the following two observations.

Observation 3. *For any given $\kappa(\Gamma)$, whether or not the two constraints* (*i*) *and* (*ii*) *in Eq.* (48) *are satisfied is completely determined by the sets $\mathcal{P}_{I_A}$ and $\mathcal{E}_{I_A}$, and the causal order on $\mathcal{P}_{I_A} \cup \mathcal{E}_{I_A}$.*

Observation 4. *For all $\kappa(\Gamma)$ that satisfy the constraints (i) and (ii) in Eq. (48), all O_X, $X \in \mathcal{X}$, are in the causal past or elsewhere of I_A.*

The proof of observation 3 is given below. Observation 4 follows from $A_O \not\preceq X_I$ and the closed laboratory assumption, which imply that $I_A \not\preceq O_X$.

From the two observations 3 and 4, it follows that the probability in Eq. (45) can be obtained by summing over various probabilities which are each independent of i_A according to Eq. (16) in the main text. Namely, to obtain the probability in Eq. (45), one sums the probability in Eq. (16) over all $\mathcal{Y}$, $\mathcal{Z}$ and $\kappa(\mathcal{Y} \cup \mathcal{Z})$ which are such that the constraints (i) and (ii) in Eq. (48) are indeed satisfied, and marginalises over all $O_X \in \mathcal{Y} \cup \mathcal{Z}$ with $X \notin \mathcal{X}$ (so that precisely the O_X with $X \in \mathcal{X}$ remain). □

In the following, we prove Observation 3 used above.

Proof of Observation 3. In the constraint (i), $A_O \not\preceq X_I$ can equivalently be replaced by $I_A \not\preceq X_I$, and $A_O \prec \Lambda_I^{(1)}$ can equivalently be replaced by $I_A \prec \Lambda_I^{(1)}$ due to the closed laboratory assumption. Also, in the constraint (i), we may without loss of generality take all $\Lambda_O^{(i)}$ and $\Lambda_I^{(i+1)}$ with $1 \leq i \leq M-1$ (and $\Lambda_O^{(M)}$) to be in the causal past or elsewhere of I_A. Namely, whenever constraint (i) is violated for some $\Lambda^{(1)}, \ldots, \Lambda^{(M)}$, and $I_A \prec \Lambda_I^{(j+1)}$ for some j with $1 \leq j \leq M-1$ (or $I_A \prec \Lambda_O^{(j)}$, which implies $I_A \prec \Lambda_I^{(j+1)}$ by transitivity), the constraint is also violated for $\Lambda^{(j+1)}$, …, $\Lambda^{(M)}$, i.e., we can "skip" $\Lambda^{(1)}$, …, $\Lambda^{(j)}$ (and if $I_A \prec \Lambda_O^{(M)}$, we would have $I_A \prec X_I$ and thus $A_O \prec X_I$ due to the closed laboratory assumption). Therefore, whether $\kappa(\Gamma)$ satisfies the constraint (i) or not is completely determined by what variables are contained in $\mathcal{P}_{I_A}$ and $\mathcal{E}_{I_A}$, and the causal order on the subset $\mathcal{P}_{I_A} \cup \mathcal{E}_{I_A}$.

Furthermore, if constraint (i) holds, the right-hand side of the constraint (ii) (i.e. the part in the second square bracket) can only be true if all variables X_O, Y_I and $\Lambda_I^{(i)}$, $\Lambda_O^{(i)}$ for $1 \leq i \leq M$ considered there are in the causal past or elsewhere of I_A (otherwise, we would have $A \prec Y$). Therefore, whether this right-hand side is true or not—and thus, whether $\kappa(\Gamma)$ satisfies the constraint (ii) or not—is also completely determined by $\mathcal{P}_{I_A}$ and $\mathcal{E}_{I_A}$, and the causal order on $\mathcal{P}_{I_A} \cup \mathcal{E}_{I_A}$. □

For completeness, we recall here a simpler characterisation of causal correlations, which is as follows [9, 10].

Causal correlation (alternative characterisation [9, 10]). *For one local experiment (i.e. $\Delta = \{A\}$), any correlation $P(o_A|i_A)$ is causal. For multiple local experiments, a correlation $P(o_A, o_B, o_C, \ldots|i_A, i_B, i_C, \ldots)$ is causal if and only if it can be decomposed as*

$$P(o_A, o_B, o_C, \ldots|i_A, i_B, i_C, \ldots) = \sum_{X\in\Delta} q_X \, P_X(o_X|i_X) \, P_{X,i_X,o_X}(o_{\Delta\backslash\{X\}}|i_{\Delta\backslash\{X\}}), \tag{49}$$

with $q_X \geq 0$, $\sum_{X\in\Delta} q_X = 1$, where (for each X) $P_X(o_X|i_X)$ is a single-partite (and hence causal) correlation and (for each X, i_X, o_X) $P_{X,i_X,o_X}(o_{\Delta\backslash\{X\}}|i_{\Delta\backslash\{X\}})$ is a causal correlation for the parties in $\Delta\backslash\{X\}$.

This characterisation can be intuitively interpreted as describing an iterative "unravelling" of the local experiments in a sequence, where, with some probability, one local experiment occurs first, then, according to some probability which depends on the setting and outcome of this first local experiment, another local experiment occurs second, and so on [9, 10].

SUPPLEMENTARY NOTE 6—WHICH ASSUMPTIONS ARE VIOLATED?

The conclusion that in an experiment that admits a description in terms of standard causal evolution in spacetime there exist physical variables which violate causal inequalities without manifestly violating the closed laboratory and free choice assumptions naturally raises the question of whether we could gain further insights into the way the causal inequality assumptions are violated. Is it possible that, in spite of the outlined considerations about the observable causal relations between the concerned variables, upon a closer inspection of the circuit in Fig. 5 we would find a sense in which the free choice or closed laboratory assumption is violated for these variables, rather than the existence of causal order *per se*? In particular, the circuit describes a sequence of interactions between sets of systems that one may intuitively associate with the three different parties. This seems to violate the closed laboratory assumption, which essentially stipulates that each party is involved in a single round of information exchange, where they receive information about the past through the input system X_I and subsequently send out information into the future through the output system X_O.

It is crucial to realise that the causal inequality assumptions concern concrete variables, which in our case we have explicitly specified. As we will see, these variables are not the same as what one might intuitively assume if one thinks of this experiment as involving three laboratories existing through time that exchange information with each other (the parties Alice, Bob, and Charlie that operate on the time-delocalised process must be understood abstractly as agents who control the parameters that describe the operations taking place on the time-delocalised systems). The possibility of understanding the experiment in terms of other variables for which a causal order exists and for which the closed laboratory assumption might appear to be violated is not in contradiction with the claim that no such interpretation is available for the variables of interest. To investigate whether such an interpretation is available for these variables, first note that any claimed causal order on the variables should have operational grounds—otherwise it is always possible to imagine some fictitious causal order and a violation of the free choice or closed laboratory assumptions relative to it so as to "explain" the observed correlations. In the present scenario, we are unaware of any other operationally grounded notion of causal order that one could invoke apart from the one imposed by spacetime, which could be further constrained by the lack of physical interaction between specific variables at different times (as in when a laboratory is kept "closed" between the time of input and the time of output). We will therefore focus on the question of whether there could exist a compelling interpretation in which the variables we have identified can be seen as taking place at definite, although possibly random and dependent on other variables, spacetime locations such that the free choice or closed laboratory assumption is violated. We will argue that no such interpretation is supported by the spatiotemporal description of the experiment (Fig. 5). On the contrary, a careful analysis of the link between the time-delocalised variables of interest and the time-local variables in the circuit leads to the conclusion that the time-delocalised variables we have identified cannot be interpreted as taking place at definite locations in the background spacetime, which is what the causal inequality violation witnesses.

First, observe that the free choice assumption is trivially compatible with the structure of the circuit in Fig. 5: the variables I_X can all be defined at the initial time, so irrespectively of what spacetime locations we may attribute to the remaining variables (which would all be in the future), there is no reason why these variables could not be chosen freely. (Of course, no matter how we choose these variables in practice, one can never exclude the in-principle possibility that there is a hidden common cause for these variables and some of the other relevant variables, but this conspiratorial possibility is trivially always present and is obviously not suggested by the circuit.)

To discuss the plausibility of the closed laboratory assumption, we need to first identify a reasonable candidate causal order on the variables, since this assumption is formulated in terms of a causal order. For the operations of Alice and Bob, there is a natural interpretation of the experiment as describing the occurrence of these operations at definite spacetime locations since these operations effectively take place within a "classical switch"—each can be thought of as taking place at one of two possible times determined by the state of a control bit. This is compatible with the time-delocalised input and output variables A_I, A_O, B_I, B_O (see Supplementary Note 4 B), which effectively reduce to the respective time-local input and output variables conditionally on the state of the control bit. As each of these effective operations is a standard time-localised operation from an input to an output system, which involves the ancillas of the respective party and constitutes the only interaction with those ancillas, the closed laboratory assumption for the respective party is manifestly respected.

The operation U_C of Charlie, however, is delocalised in time in a different way, which can intuitively be described as follows: the operation either happens at the beginning of the circuit (in the case when the controlled operation in the last stage is not triggered and hence that controlled operation can be effectively omitted), or it happens at the very end of the circuit (when the controlled operation in the last stage is triggered and it effectively results in "undoing" the effect of the first U_C (by the action of $U_C^\dagger$) followed by applying the NOT gate σ_{x} and then U_C anew). Notice that if we interpret the operation of Charlie in this way, it is again a standard operation taking place at one of two possible times that manifestly respects the closed laboratory assumption. How is it then possible that we obtain a causal inequality violation?

The answer is that the interpretation just outlined makes no operational sense, which also transpires in the fact that it contradicts the causal order of events in spacetime. Indeed, if we think that the operation of Charlie takes place at one of the two possible times, we must conclude that whether it happens at the earlier time or not (which is a variable associated with that time) depends on the state of the controlled bits at the end, which itself can be influenced by the operations of Alice and Bob. This would amount to influence by Alice and Bob on the past, which is in contradiction with spacetime causality. The error leading to this contradiction is in not recognizing that in order to say that a given variable such as C_I or C_O "takes place" at a given time, it must in principle be possible for an agent at the same time to know with certainty whether this is the case. This is clearly not possible at the first of the two candidate temporal locations for the operation U_C since the state of the control bits is not yet known at that time. This is in contrast to the situation for U_A or U_B whose time depends on a variable in the past that can be known at the time of the operation.

An analogous problem arises if one attempts any other obvious "localisation" of these variables, such as for instance the one suggested by the isomorphism in Supplementary Fig. 10. In that case, C_I could be thought of as effectively corresponding to P_3 at the initial time but up to a NOT gate that is controlled by variables in the future. Associating C_I with the initial time would, however, be in contradiction with spacetime causality and, as before, makes no sense since C_I cannot be known at that time.

We now provide a general proof in the case when the process is treated as quantum, that the quantum input system C_I cannot be effectively associated with definite times in principle. The operations of Alice and Bob will be interpreted as localised in time conditionally on the computational basis of Q_1 as discussed previously.

Consider the case where each of the unitaries U_A and U_B is a SWAP operation (sending X'_I to X_O and X_I to X'_O, $X = A, B$), and U_C is the identity channel from C_I to C_O (hence no explicit ancilla for Charlie needs to be considered). This situation is depicted in Supplementary Fig. 15.

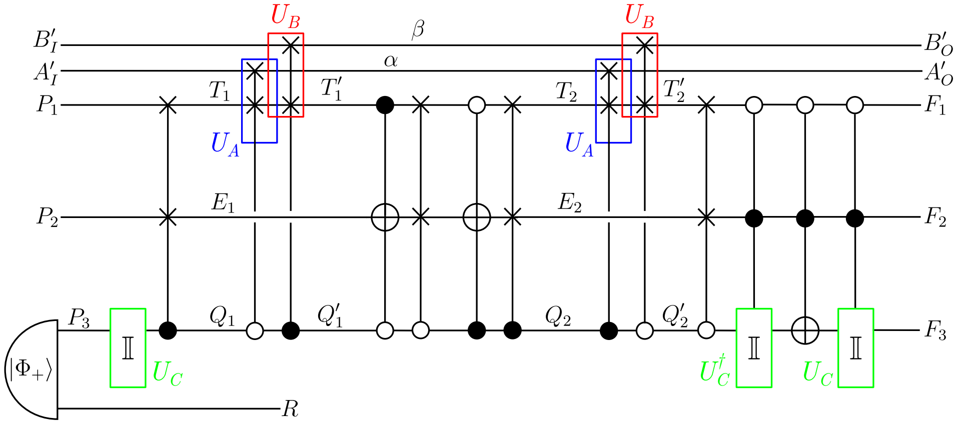

Supplementary Fig. 15. Temporal circuit of Fig. 5, with SWAP operations for Alice and Bob, an identity channel for Charlie and an additional, time-local "reference" system R. The systems P_3 and R are prepared in a maximally entangled state $|\Phi_+\rangle$ at the beginning of the circuit.

As depicted in the figure, we also introduce an additional, time-local, "reference" system R at the beginning of the circuit. Since R is separate from C_I, the joint Hilbert space of R and C_I is $\mathcal{H}^{C_I R} = \mathcal{H}^{C_I} \otimes \mathcal{H}^R$. (Note that arbitrary preparations and measurements could be applied on the system $\mathcal{H}^{C_I} \otimes \mathcal{H}^R$ by trivially extending the previously described procedures for C_I onto R, hence this is an operationally meaningful Hilbert space.) Let the joint system P_3R be initially prepared in the state $|\Phi_+\rangle = (|00\rangle + |11\rangle)/\sqrt{2}$ (Supplementary Fig. 15). Considering the precise definition of C_I through the isomorphism in Supplementary Fig. 10(a), we will now argue that C_I cannot be consistently associated with any definite time since no quantum system at a definite time contains the correct information implied by this definition.

Let us restrict our attention to the cases where the systems A'_I and B'_I are each prepared in one of the computational basis states $|0\rangle$ or $|1\rangle$. Through the SWAP operations performed by Alice and Bob, these states are transferred onto A_O and B_O, respectively. Now, according to the isomorphism in Supplementary Fig. 10(a), for any such combination of these states, which could be freely chosen by Alice and Bob, the state on the system C_I reduces to the state of P_3, or to the state of P_3 up to a NOT gate σ_x. This property holds for the full operator algebras on the respective systems and thus remains true also when we regard C_I and P_3 as parts of larger systems that include R: depending on the computational basis states output by Alice and Bob, C_IR reduces to P_3R or P_3R up to a NOT gate on P_3. In our case, this means that the state on C_IR is either $(|00\rangle + |11\rangle)/\sqrt{2}$ or $(|10\rangle + |01\rangle)/\sqrt{2}$. Furthermore, by choosing the combination of basis states that they output, Alice and Bob can fully determine which of these two orthogonal

states is received on $C_I R$. But since these two states are perfectly distinguishable, this means that we have perfect signalling from A'_I and B'_I to $C_I R$. This, in turn, means that C_I could not possibly exist prior to the time of the controlled operations of Alice and Bob in the circuit, even with small nonzero probability (unless it is defined as a subsystem that overlaps with A'_I and B'_I, which however would contradict the premise that it is a subsystem separate from A'_I and B'_I). Indeed, let us assume that with some nonzero probability C_I can be associated with such prior times. For the same reasons as explained in the previous examples, whether this is the case or not cannot depend on the choices of Alice and Bob that are only used in the future, since whether the variables "take place" at a given time must be possible to know at the respective time. But then in the hypothetical cases in which C_I takes place prior to the operations of Alice and Bob, Alice and Bob could make different choices altering the state of $C_I R$, which is in contradiction with spacetime causality. Therefore, C_I could not be associated with any time prior to the operations of Alice and Bob.

We will now show that C_I cannot be consistently associated with only later times either. Let us consider the case in which A'_I is prepared in the state $|1\rangle$, B'_I in the state $|0\rangle$, P_1 in the state $|0\rangle$ and P_2 in the state $|1\rangle$. In this case, it is straightforward to verify that the controlled operations at the end of the circuit are not triggered and the state on $C_I R$ is $(|00\rangle + |11\rangle)/\sqrt{2}$. Since the state of R is purified on C_I, if C_I exists at any time after the beginning of the operations of Alice and Bob, it must be possible to find the purification of R at this time. However, if we track this information in time, we see that it is "lost" as soon as the first controlled operation of Alice or Bob happens. Indeed, following the evolution of the computational basis it is straightforward to verify that the controlled-SWAP gate immediately after the first gate U_C correlates R with T_1 in the computational basis, and this correlation is propagated onto the ancillas of Alice and Bob by the first controlled operations of Alice and Bob, remaining there until the end of the circuit. (Note that for the natural time localisation of the operations of Alice and Bob assumed here, the ancillary wire α coincides with A'_I or A'_O depending on the time at which U_A takes place, and similarly β coincides with B'_I or B'_O. Since by definition C_I is separate from these ancillary systems, it cannot overlap with them.)

To summarise, we have shown that the time-delocalised quantum subsystem C_I that we have identified cannot be effectively localised in time, since there exists no time-local subsystem that contains the correct information as per the definition of C_I. For this argument, it was essential that we treated the system as a quantum system as we used the property that quantum information cannot be copied in order to argue that no subsystems could exists that contains the required quantum information at any given time. This argument does not automatically imply that the classical variable corresponding to the computational basis of C_I could not be effectively localised. Indeed, in the example we considered, a copy of this variable remains available through time and thus this variable in principle could be declared associated with the future. (As in the quantum case, we can rule out the possibility that this variable takes place in the past even with small probability since Alice and Bob can fully determine its value.) However, if we assume that the classical variable C_I, however localised in time, should not overlap with any ancillary systems that could be introduced for Charlie, we can easily perform a version of the quantum argument leading to the same conclusion: we now introduce an ancilla for Charlie and let Charlie perform a SWAP operation, which already at the start of the circuit transfers the value of P_3 onto this ancilla (see Supplementary Fig. 16). This makes it impossible to find a system that contains the correct information expected to live on C_I at later times only, except if that subsystem is defined to overlap with the ancillas.

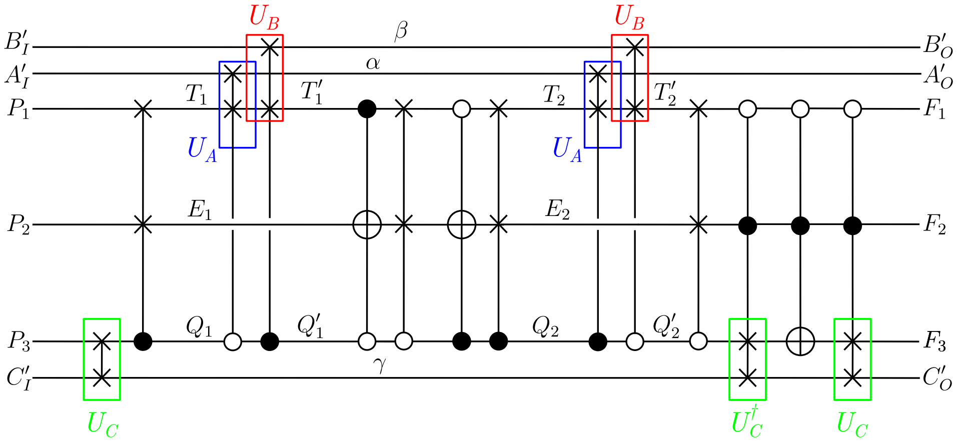

Supplementary Fig. 16. Temporal circuit of Fig. 5, with SWAP operations for Alice, Bob and Charlie.

In conclusion, we have shown that some of the time-delocalised variables that we have identified do not admit an effective localisation in time by showing that there do not exist time-local variables that take their value. This

prevents us from assigning a definite causal order on all variables, which is arguably what the violation of the causal inequality witnesses. Our argument for the purely classical case is somewhat weaker than the quantum case as it assumes that the variable C_I does not live on the intermediate ancillary wire with which Charlie is made to interact. While this seems intuitive, this assumption could in principle be debated since the intermediate ancillary wire of Charlie need not coincide with C'_I or C'_O, which must be separate from C_I by definition. It would be interesting to investigate whether this assumption could be relaxed as in the quantum case.

[1] O. Oreshkov, Quantum **3**, 206 (2019), arXiv:1801.07594 [quant-ph].
[2] G. Chiribella, G. M. D'Ariano, and P. Perinotti, EPL **83**, 30004 (2008), arXiv:0804.0180 [quant-ph].
[3] G. Chiribella, G. M. D'Ariano, and P. Perinotti, Phys. Rev. A **80**, 022339 (2009), arXiv:0904.4483 [quant-ph].
[4] G. Gutoski and J. Watrous, in *Proceedings of 39th ACM STOC* (2006) pp. 565–574, arXiv:quant-ph/0611234.
[5] J. Barrett, R. Lorenz, and O. Oreshkov, Nat. Commun. **12**, 885 (2021), arXiv:2002.12157 [quant-ph].
[6] W. Yokojima, M. T. Quintino, A. Soeda, and M. Murao, Quantum **5**, 441 (2021), arXiv:2003.05682 [quant-ph].
[7] J. Wechs, H. Dourdent, A. A. Abbott, and C. Branciard, PRX Quantum **2**, 030335 (2021), arXiv:2101.08796 [quant-ph].
[8] P. A. Guérin and Č. Brukner, New J. Phys. **20**, 103031 (2018), arXiv:1805.12429 [quant-ph].
[9] O. Oreshkov and C. Giarmatzi, New J. Phys. **18**, 093020 (2016), arXiv:1506.05449 [quant-ph].
[10] A. A. Abbott, C. Giarmatzi, F. Costa, and C. Branciard, Phys. Rev. A **94**, 032131 (2016), arXiv:1608.01528 [quant-ph].
